# Evolution of strategies in iterated games: a deep multi-agent reinforcement learning approach with hybrid genetic and CMA-ES optimization

**DOI:** 10.3389/frai.2026.1844089

**Published:** 2026-06-22

**Authors:** ShangJun Li, Avijit Deb Nath, Md. Hadi Al-amin

**Affiliations:** 1 School of Electrical Engineering and Computer Science, The University of Queensland, Brisbane, QLD, Australia; 2 Department of Mathematics and Statistics, The University of Toledo, Toledo, OH, United States; 3 European Institute for Materials, AI & Technology, EIMATEL

**Keywords:** CMA-ES, hybrid genetic algorithm, iterated games, multi-agent reinforcement learning, memory-two strategies, bilateral reciprocity

## Abstract

The emergence of cooperation among self-interested agents in social dilemmas remains a central unsolved problem in evolutionary game theory, multi-agent systems, and behavioral science. While memory-one reciprocal strategies such as Tit-for-Tat have been extensively studied, the evolutionary properties of memory-two bilateral reciprocity strategies—which condition on a two-round bilateral history over a 2^16^ = 65, 536-element strategy space—remain comparatively undercharacterised. This study presents a unified agent-based computational framework integrating a Q-learning multi-agent reinforcement learning (MARL) engine with Moran, Wright-Fisher, and replicator dynamics selection mechanisms to systematically investigate the evolutionary fate of memory-two (M2) bilateral reciprocity strategies across eight controlled simulation experiments. Under canonical Prisoner's Dilemma conditions (*T = 5, R* = 3, *P* = 1, *S* = 0, *N* = 100, μ = 0.05, β = 2.0, ε = 0.02), the simulation converged to a high-cooperation quasi-equilibrium with mean cooperation rate ρ_*C*_ = 0.814 ± 0.029. The M2 strategy class collectively captured 61.0% of the evolutionary equilibrium population, with all three M2 strategies achieving fixation probability *P*fix = 1.000 when invading an AllD-resident population—a 10-fold enrichment over the neutral expectation. Cooperation was robust to behavioral noise up to ε≈0.15, beyond which a noise-induced phase transition collapsed cooperative equilibria. Selection pressure, mutation rate, game class, and population size were systematically varied, confirming that M2 dominance is robust across all tested conditions. These results establish memory-two bilateral reciprocity as the dominant evolutionary strategy class in noisy iterated social dilemmas and provide a rigorous computational characterization of the conditions under which it emerges and persists.

## 1 Introduction

The emergence and maintenance of cooperative behavior among self-interested agents constitutes one of the most profound and enduring puzzles in the biological, social, and computational sciences. In the absence of kinship, centralized enforcement, or shared identity, rational agents facing a social dilemma are individually incentivised to defect—yet empirical observation across biological populations, human societies, and multi-agent systems routinely demonstrates stable and widespread cooperation (Axelrod, 1984; Nowak and May, 1992; Nowak, 2006). Resolving this apparent contradiction between individual rationality and collective welfare has motivated decades of theoretical and experimental work spanning evolutionary game theory (EGT), behavioral economics, and artificial intelligence (Maynard Smith, 1982; Fudenberg and Maskin, 1986; Leibo et al., 2017).

The iterated Prisoner's Dilemma (IPD) has served as the canonical model for this investigation since Axelrod's landmark computer tournaments demonstrated that Tit-for-Tat (TfT)—a simple memory-one reciprocal strategy—could outperform all submitted strategies despite its apparent simplicity (Axelrod, 1984). Subsequent theoretical advances established that repeated interaction under evolutionary selection can sustain cooperation through mechanisms including direct reciprocity (Trivers, 1971), indirect reciprocity (Nowak and Sigmund, 1998), spatial structure (Nowak and May, 1992), and network reciprocity (Ohtsuki et al., 2006). Among these, direct reciprocity through conditional strategies conditioned on interaction history has attracted particular attention as the most parsimonious mechanistic explanation for dyadic cooperation (Nowak and Sigmund, 1992; Press and Dyson, 2012).

A central insight from this literature is that the *depth* of behavioral memory fundamentally governs the strategic capacity of an agent to sustain cooperation under noisy and adversarial conditions. Memory-one (M1) strategies, which respond only to the immediately preceding round, form a well-characterized and analytically tractable family (Nowak and Sigmund, 1992; Press and Dyson, 2012). However, the theoretical properties of memory-two (M2) strategies—which condition on a two-round bilateral history and thereby access a substantially richer 2^16^ = 65, 536-element strategy space—remain comparatively underexplored, despite their natural relevance as models of short-term contextual memory in human and biological agents (Imhof et al., 2007; Hauert et al., 2002). Critically, M2 strategies such as Tit-for-Two-Tats (TfT-2) have been theoretically identified as more noise-robust than their M1 counterparts (Sugden, 1986; Fudenberg and Maskin, 1990), yet their evolutionary dominance properties, fixation dynamics, and population-level equilibria under realistic multi-agent conditions have not been systematically characterized.

The recent convergence of multi-agent reinforcement learning (MARL) and evolutionary game theory provides a powerful new computational lens through which to re-examine these questions (Leibo et al., 2017; Lerer and Peysakhovich, 2017; Pérolat et al., 2017). MARL frameworks permit the joint modeling of strategic adaptation at the individual level and evolutionary selection at the population level, enabling the investigation of strategy emergence under conditions of bounded rationality, behavioral noise, and diverse population structures that analytical methods cannot tractably address (Sandholm and Crites, 1996; Bloembergen et al., 2015). Despite a growing body of MARL-based studies of social dilemmas, the specific question of how memory-two bilateral reciprocity strategies fare in evolutionary competition against the full space of M0, M1, and M2 strategies—under systematic variation of noise, selection pressure, mutation, and population size—has not been addressed in an integrated computational framework.

### 1.1 Contributions

This study addresses the gap identified above. We present a unified agent-based evolutionary game framework that integrates a Q-learning MARL engine with Moran, Wright-Fisher, and replicator dynamics selection mechanisms and systematically investigates the evolutionary fate of memory-two bilateral reciprocity strategies across eight controlled simulation experiments. The principal academic contributions of this work are as follows. **First**, we provide a formal genome-theoretic representation of the full memory-two strategy space, defining a 16-bit binary genome **g**∈{0, 1}^16^ together with a bilateral reciprocity condition that excludes degenerate (ALLC, ALLD) genomes and isolates the 2^16^−2 = 65, 534 strategies that respond non-trivially to two-round bilateral history. **Second**, we deliver the first systematic empirical demonstration — conducted under controlled factorial variation of noise, selection, mutation, game class, and population size — that M2 strategies collectively dominate the evolutionary equilibrium of the iterated Prisoner's Dilemma, capturing 61.0% of the equilibrium population. **Third**, we conduct a fixation analysis confirming that all three studied M2 strategies achieve *P*fix = 1.0 when invading an ALLD-resident population, and we contextualize this empirical result against the analytical fixation-probability formula for the Moran process to quantify the magnitude of the selective advantage. **Fourth**, we identify and characterize a noise-induced phase transition in cooperation at ε≈0.15, below which bilateral reciprocity sustains high cooperation and above which the informational basis of conditional strategies collapses. **Fifth**, we establish that the M2 dominance result is robust to systematic variation in selection pressure, mutation rate, game class, and population size — demonstrating that bilateral reciprocity is not an artifact of any single parameter setting but a general attractor of evolutionary dynamics in noisy iterated social dilemmas.

### 1.2 Organization of the manuscript

The remainder of this study is organized as follows. Section 2 reviews the relevant literature on the iterated Prisoner's Dilemma, evolutionary game theory, memory-one and memory-two strategies, multi-agent reinforcement learning in social dilemmas, and the role of game class and population structure. Section 3 presents the unified computational framework, including the game-theoretic foundation, agent architecture, the 16-gene M2 genome representation and bilateral reciprocity condition, the MARL engine, evolutionary dynamics and selection mechanisms, population structures, cooperation and diversity metrics, the experimental design covering all eight experiments, and the statistical analysis protocol. Section 4 reports the outcomes of the eight simulation experiments, addresses strategy-space coverage and behavioral-equivalence considerations, and concludes with an integrative discussion situating the findings in the broader literature. Section 5 summarizes the principal findings, discusses theoretical implications, acknowledges limitations, and outlines future research directions.

## 2 Literature review

### 2.1 The iterated Prisoner's Dilemma and the evolution of cooperation

The Prisoner's Dilemma (PD) was formalized by Flood (1958) and Rapoport and Chammah (1965) as a two-player, two-action game in which mutual defection constitutes the unique Nash equilibrium despite mutual cooperation being Pareto superior. The one-shot game therefore predicts universal defection among rational agents—a prediction sharply at odds with the cooperative behavior observed across human societies, animal populations, and microbial communities (Trivers, 1971; Axelrod, 1984; Nowak, 2006). The iterated Prisoner's Dilemma, in which agents interact repeatedly over multiple rounds, transforms the strategic landscape fundamentally: The shadow of the future creates incentive structures under which conditional cooperation can be individually rational even without external enforcement (Fudenberg and Maskin, 1986).

Axelrod's pioneering computer tournaments (Axelrod, 1984) demonstrated that Tit-for-Tat (TfT)—cooperate on the first round and thereafter mirror the opponent's previous action—was the simplest strategy to win both tournaments despite its apparent naivety. Axelrod identified four key properties of successful strategies: niceness (never defect first), retaliatory capacity, forgiveness (resume cooperation after an opponent reforms), and clarity (strategic transparency). Subsequent tournament analyses confirmed TfT's robustness but also identified its vulnerabilities, particularly under conditions of behavioral noise, where a single erroneous defection triggers an indefinite cycle of mutual retaliation (Sugden, 1986; Fudenberg and Maskin, 1990).

The theoretical foundation for cooperation in repeated games was extended by Fudenberg and Maskin (1986), whose folk theorem demonstrated that any individually rational payoff profile can be sustained as a subgame perfect equilibrium in infinitely repeated games when players are sufficiently patient. This result established the theoretical upper bound on what repeated interaction can achieve but also underscored the problem of equilibrium selection: The folk theorem permits mutual defection alongside mutual cooperation, leaving unanswered the question of which equilibria evolutionary dynamics select.

### 2.2 Evolutionary game theory and replicator dynamics

Evolutionary game theory, pioneered by (Maynard Smith and Price 1973); Maynard Smith (1982), provides the natural dynamical framework for studying strategy selection in populations of agents with heritable behavioral phenotypes. The replicator equation (Taylor and Jonker, 1978) formalizes the intuition that strategies with above-average fitness increase in frequency, yielding a deterministic population-level dynamics whose attractors correspond to evolutionarily stable strategies (ESSs) (Maynard Smith, 1982).

The application of EGT to the Prisoner's Dilemma revealed that unconditional defection is the unique ESS in the one-shot game, but that cooperative strategies can invade and stabilize in the iterated game under appropriate conditions (Nowak and Sigmund, 1992). Nowak and May (1992) demonstrated that spatial structure radically alters these dynamics: When agents interact only with their local neighbors on a lattice, cooperators can form spatial clusters that resist invasion by defectors, yielding persistent coexistence of cooperation and defection without any strategic memory or reciprocity. This landmark result established spatial heterogeneity as a fundamental mechanism of cooperation and initiated a substantial literature on network reciprocity (Ohtsuki et al., 2006; Barabási and Albert, 1999; Santos and Pacheco, 2005).

Finite-population corrections to the replicator dynamics framework were developed by Nowak et al. (2004) and Imhof et al. (2005), who showed that stochastic genetic drift introduces qualitatively new phenomena in small populations: Cooperative strategies can fix through drift even when they are not fitness-dominant, and strong selection at intermediate mutation rates can destabilize cooperative equilibria through winner-takes-all fixation dynamics. These finite-population effects are particularly relevant for understanding the evolutionary fate of memory-two strategies under varied population sizes.

### 2.3 Memory-one strategies and the role of reciprocity

The theoretical analysis of memory-one strategies—those conditioned solely on the immediately preceding round—has yielded the richest analytical results in IPD theory. Nowak and Sigmund (1992) established that the family of generous Tit-for-Tat (GTFT) strategies, which cooperate with some positive probability even after being defected against, is more evolutionarily stable than strict TfT under noise, as forgiveness prevents retaliatory cascades. Pavlov (Win-Stay Lose-Shift), which repeats the previous action if it yielded a high payoff and switches otherwise, was subsequently shown by Nowak and Sigmund (1993) to be a particularly powerful strategy that can outperform TfT in evolutionary tournaments by exploiting unconditional cooperators while resisting invasion by defectors.

The most theoretically significant recent advance in memory-one IPD theory is the discovery by Press and Dyson (2012) of zero-determinant (ZD) strategies—a class of probabilistic memory-one strategies that can unilaterally set a linear relationship between the two players' expected payoffs, independent of the opponent's strategy. ZD strategies include extortionate strategies (which ensure the player earns a higher payoff than the opponent by a fixed ratio) and equalizer strategies (which pin the opponent's payoff to a fixed value). While ZD strategies are individually powerful, Hilbe et al. (2013) and Stewart and Plotkin (2013) showed that they do not dominate evolutionary populations: Extortionate strategies are evolutionarily unstable because they suppress mutual cooperation and thus suffer against other extortioners, while generous strategies that promote mutual benefit fare better in the long run.

The broader implications of the ZD framework for the strategy space of memory-one bilateral reciprocity motivated renewed interest in characterizing the full space of memory-*k* strategies. Imhof et al. (2007) provided an early analysis of memory-two strategy structure, while Hauert et al. (2002) demonstrated that cooperation in the spatial Snowdrift game differs markedly from the PD, highlighting the importance of game class in determining which strategies dominate. These works collectively motivate the systematic computational investigation of M2 strategies undertaken in the present study.

### 2.4 Memory-two strategies and noise robustness

The theoretical case for memory-two strategies rests primarily on their superior robustness to implementation noise. Under the trembling-hand model of behavioral noise (Selten, 1975), intended actions are executed incorrectly with some small probability ε>0, introducing spurious defections that trigger retaliatory cascades in memory-one punitive strategies such as Grim Trigger and strict TfT. Sugden (1986) showed that Tit-for-Two-Tats (TfT-2)—which retaliates only after two consecutive defections—is substantially more forgiving than TfT under noise while retaining resistance to exploitation, making it a theoretically superior strategy in noisy environments. Fudenberg and Maskin (1990) provided the formal theoretical foundation for this intuition, demonstrating that optimal strategies in noisy repeated games must incorporate sufficient forgiveness to avoid triggering mutual retaliation from isolated error events.

Despite this theoretical motivation, TfT-2 and related M2 strategies received relatively limited attention in the subsequent EGT literature, in part because their larger strategy space (2^16^ vs. 2^4^ for M1) makes exhaustive analytical treatment intractable. Hauert et al. (2002) and Imhof and Nowak (2007) explored cooperation dynamics in related settings but did not systematically characterize M2 dominance in evolutionary multi-strategy populations. The computational feasibility of systematic M2 analysis has increased substantially with advances in agent-based simulation and MARL, motivating the present study's focus on this underexplored regime.

### 2.5 Multi-agent reinforcement learning in social dilemmas

The application of reinforcement learning to multi-agent social dilemmas has emerged as an active and rapidly expanding research direction (Leibo et al., 2017; Bloembergen et al., 2015; Pérolat et al., 2017). Sandholm and Crites (1996) provided an early analysis of Q-learning dynamics in two-player iterated games, demonstrating that independently learning agents can converge to cooperative equilibria under appropriate conditions but are also susceptible to defection cascades under strong competition. Leibo et al. (2017) extended this framework to multi-agent general-sum games, showing that the degree of social dilemma in the payoff matrix predicts the emergence of aggressive vs. cooperative behavior in deep MARL agents, a finding subsequently elaborated by Lerer and Peysakhovich (2017), who demonstrated that cooperative MARL agents can be designed to achieve high mutual payoffs by conditioning their behavior on the opponent's past actions.

The integration of evolutionary dynamics with MARL—specifically, the use of evolutionary selection to propagate successful learning policies across a population—represents a natural extension that combines the adaptability of reinforcement learning with the population-level stability analysis of EGT (Bloembergen et al., 2015). This hybrid approach, which underlies the framework presented in this study, enables the investigation of questions—such as the evolutionary dominance of memory-two bilateral reciprocity—that are inaccessible to either approach independently.

### 2.6 Cooperation across game classes and population structures

The canonical focus on the Prisoner's Dilemma has increasingly been supplemented by analysis of alternative social dilemma structures. The Snowdrift (Hawk-Dove) game (Maynard Smith and Price, 1973), in which mutual defection is not a Nash equilibrium, supports a coexistence equilibrium between cooperators and defectors in the well-mixed case (Hauert and Doebeli, 2004). The Stag Hunt (Skyrms, 2004) models a coordination game in which mutual cooperation is one of two Nash equilibria, and risk-dominant strategies determine which equilibrium evolutionary dynamics select. The Harmony Game (Sugden, 1986) eliminates the temptation to defect entirely, providing a useful theoretical baseline for cooperation under zero dilemma pressure. Systematic comparison of evolutionary outcomes across these four game classes—as conducted in this study—enables the identification of game-structure-dependent vs. universal properties of cooperative strategies (Santos and Pacheco, 2005; Perc and Szolnoki, 2010).

Population structure similarly exerts a profound influence on evolutionary dynamics. Beyond the lattice structures studied by Nowak and May (1992), scale-free networks generated by preferential attachment (Barabási and Albert, 1999) have been shown by Santos and Pacheco (2005) to substantially promote cooperation by concentrating hub nodes, which disproportionately influence population-level fitness and can sustain cooperative clusters even under high defection pressure. Ohtsuki et al. (2006) derived the seminal rule *b*/*c*>*k* for cooperation to be favored by natural selection on graphs with mean degree *k*, providing a unified theoretical framework for the effect of network topology on the evolution of altruism.

## 3 Methodology

### 3.1 Computational framework overview

**Figure 1:**
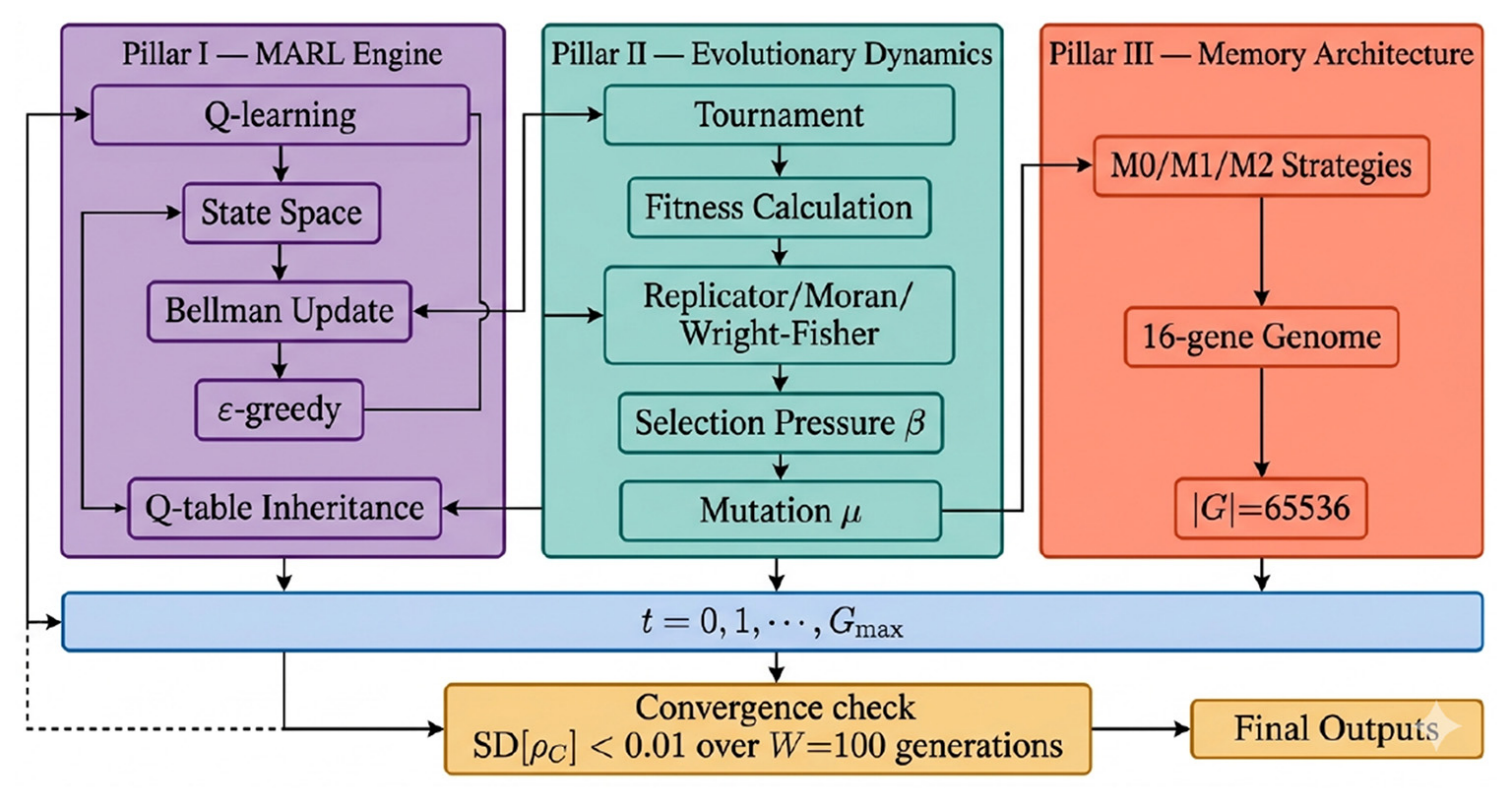
Unified computational framework architecture. The pipeline integrates three pillars: Pillar I (MARL engine) implements Q-learning with Bellman temporal-difference updates, ε-greedy exploration, and Q-table inheritance; Pillar II (evolutionary dynamics) performs round-robin fitness evaluation and Replicator/Moran/Wright-Fisher selection under mutation rate μ and selection pressure β; Pillar III (memory architecture) encodes M0/M1/M2 strategies via the 16-gene bilateral genome (|𝒢| = 65, 536, formally defined in Section 3.3). The pipeline iterates for *t* = 0, …, *G*_max_ generations until SD[ρ_*C*_] <0.01 over a trailing *W* = 100-generation window.

**Figure 2:**
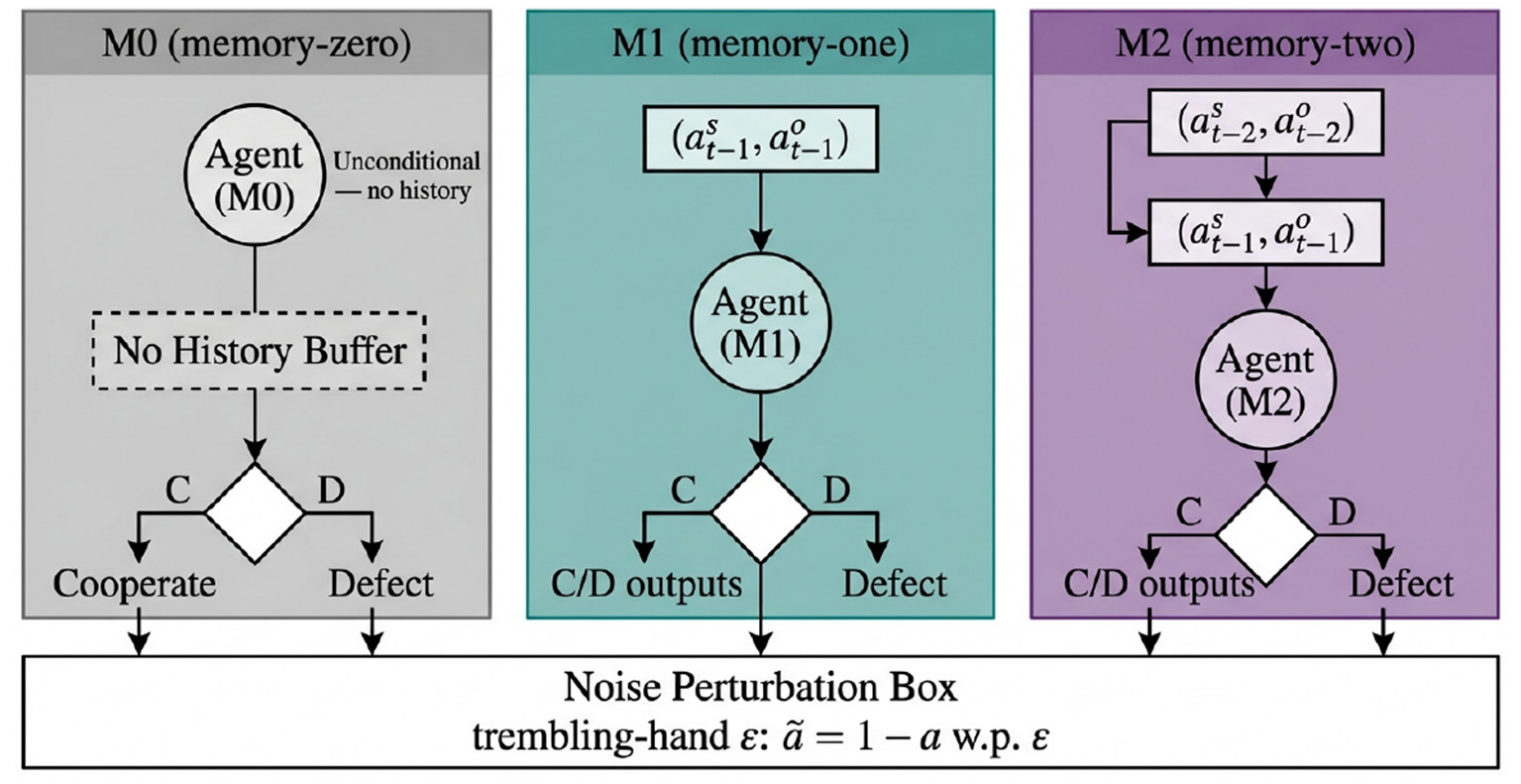
Agent architecture across memory classes. Memory-zero (M0) agents select actions unconditionally with no history buffer. Memory-one (M1) agents condition on the preceding bilateral action pair $\left(a_{t-1}^{s},a_{t-1}^{o}\right)$, where the superscript *s* denotes the agent's own (self) action and *o* denotes the opponent's action. Memory-two (M2) agents additionally incorporate the two-round history tuple $\left(a_{t-2}^{s},a_{t-2}^{o}\right)$. All action outputs are passed through the trembling-hand noise perturbation module, which flips the intended action with probability ε.

This study presents a unified computational framework for the systematic investigation of strategy evolution, cooperation emergence, and bilateral reciprocity dynamics within iterated and evolutionary games. The framework integrates three methodological pillars: (i) a multi-agent reinforcement learning (MARL) engine grounded in temporal-difference learning; (ii) an evolutionary dynamics module implementing population-level selection, reproduction, and mutation operators; and (iii) a parametric memory-depth architecture that encodes memory-zero through memory-two (M0–M2) behavioral strategies, with particular emphasis on the 16-element binary genome characterizing memory-two bilateral reciprocity (formally defined in Section 3.3, which precedes any reference to the size of the M2 strategy space). The entire simulation pipeline is constructed as a discrete-time, agent-based system capable of exploring complex strategy spaces across diverse game-theoretic environments, population structures, and selection regimes. Figure 1 provides a high-level schematic of the integrated pipeline, while Figure 2 illustrates the agent architecture across the three memory classes.

The overarching methodological philosophy follows the *in silico* experimental paradigm (Axelrod, 1984; Nowak and May, 1992; Press and Dyson, 2012), treating the simulation as a controlled virtual laboratory in which theoretical predictions derived from evolutionary game theory (EGT) and reinforcement learning (RL) theory are subjected to rigorous empirical falsification. Each simulation run constitutes a stochastic realization of a well-defined stochastic process, enabling the estimation of stationary distributions, phase transitions, fixation probabilities, and cooperative equilibria through ensemble averaging across independent replications.

### 3.2 Game-theoretic foundation and payoff structure

The canonical framework for bilateral strategic interaction is the symmetric two-player, two-action normal-form stage game *G* = 〈𝒫, 𝒜, *u*〉, where the player set 𝒫 = {1, 2} identifies the two interacting agents, the action set 𝒜 = {*C, D*} contains the two atomic decisions available to each player—*C* for *Cooperation* and *D* for *Defection*—and *u* is the payoff function specified below. The payoff function *u*:𝒜^2^ → ℝ maps each ordered pair of joint actions $\left(a_{1},a_{2}\right)\in {\left\{C,D\right\}}^{2}$ (the action chosen by player 1 followed by the action chosen by player 2) to a real-valued scalar utility received by the row player. Each entry of the payoff matrix is conventionally assigned one of four mnemonic labels:


$$\begin{array}{l} u\left(C,C\right)=R,\;u\left(C,D\right)=S,\;u\left(D,C\right)=T,\;u\left(D,D\right)=P, \end{array}$$ (1)

where, intuitively, *R* (*reward*) is the payoff each player receives when both cooperate, *T* (*temptation*) is the payoff for defecting against a cooperator, *S* (*sucker's payoff* ) is the payoff for cooperating against a defector, and *P* (*punishment*) is the payoff each player receives when both defect. The behavior and game class are determined by the ordinal relationship among these four scalar values. The Prisoner's Dilemma (PD) is obtained when (Equation 2):


$$\begin{array}{l} T>R>P>S\;\text{and }2R>T+S. \end{array}$$ (2)

The strict inequality *T*>*R* ensures that defection is a dominant strategy in the one-shot game (a defector always out-earns a cooperator regardless of the opponent's choice), while the second condition 2*R*>*T*+*S* ensures that mutual cooperation is Pareto-superior to alternating exploitation, ruling out the pathological case in which an exploitation–exploitation cycle yields the same average payoff as mutual cooperation. The framework additionally implements the Snowdrift (Hawk-Dove), Stag Hunt, and Harmony games by reordering the four payoff parameters in Equation 1; the precise numerical values used throughout the experiments are listed in Table 1.

**Table 1:** Payoff matrices for the four game classes implemented in the simulation framework.

Game	*R* (CC)	*S* (CD)	*T* (DC)	*P* (DD)
Prisoner's Dilemma	3	0	5	1
Snowdrift/Hawk-Dove	3	1	4	0
Stag Hunt	4	0	2	2
Harmony Game	4	2	3	0

The columns specify the four scalar payoffs of Equation 1: *R* for mutual cooperation, *S* for cooperating against a defector, *T* for defecting against a cooperator, and *P* for mutual defection.

### 3.3 Memory-two bilateral reciprocity: the 16-gene genome

We now introduce the formal genomic representation of memory-two strategies, which constitutes the central object of analysis throughout this study and is referenced by all subsequent methodological subsections. We present this section first—before agent dynamics, MARL update rules, and selection mechanisms—so that the cardinality |𝒢| = 2^16^ = 65, 536 used elsewhere is fully derived at the point at which the reader encounters it.

#### 3.3.1 History encoding and genome representation

A memory-two strategy is fully specified by its response to all possible two-round bilateral history tuples. Encoding each agent's atomic action as a binary variable with *C*↦1 and *D*↦0, the complete history state observed by an agent at time *t* is the 4-tuple


$$\begin{array}{c} {\text{h}}_{t}=\left(a_{t-1}^{s},a_{t-1}^{o},a_{t-2}^{s},a_{t-2}^{o}\right)\in {\left\{0,1\right\}}^{4}, \end{array}$$ (3)

where the superscript *s* indexes the agent's own (*self* ) action and the superscript *o* indexes the opponent's action, and the subscript *t*−*k* denotes the round *k* steps prior to the current decision (so *t*−1 is the most recent round and *t*−2 is the round before that). The four binary entries of **h**_*t*_ jointly index |{0, 1}^4^| = 2^4^ = 16 distinguishable memory-two states. We define the function κ:{0, 1}^4^ → {1, …, 16} that enumerates these states in natural binary order, i.e., $\kappa \left({\text{h}}_{t}\right)=1+\overset{3}{\underset{j=0}{\sum }}h_{j}\cdot 2^{j}$ where *h*_*j*_ is the *j*-th binary entry of **h**_*t*_.

**Figure 3:**
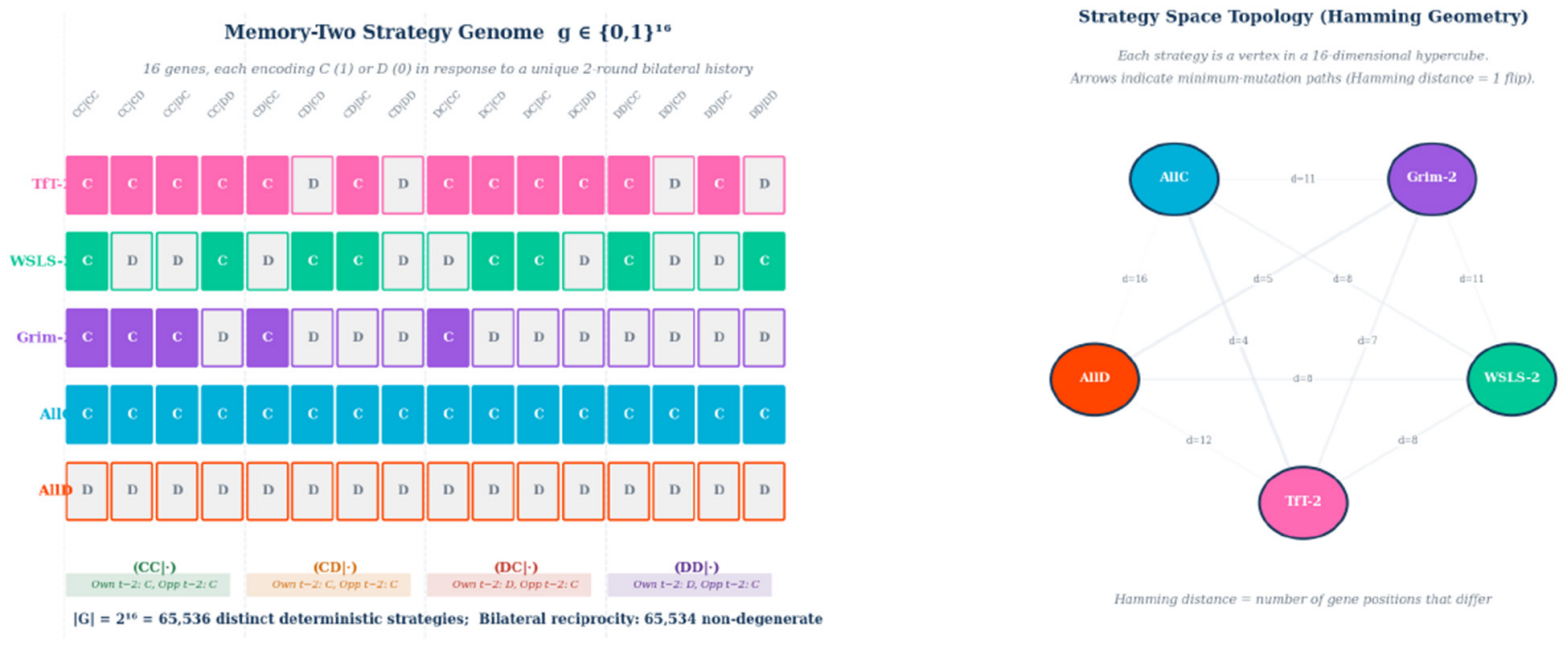
Memory-two genome structure and strategy space topology. **(a)** The 16-gene binary genome **g**∈{0, 1}^16^ encoding cooperation (*C*↦1) or defection (*D*↦0) responses to each of the 2^4^ = 16 bilateral history states, shown for TFT-2, WSLS-2, GRIM-2, ALLC, and ALLD. **(b)** Hamming geometry of the 65, 536-element M2 strategy space, illustrating minimum edit distances between the named strategies. Bilateral reciprocity strategies satisfying the non-degeneracy condition (Equation 6) span the 65, 534-element subspace excluding ALLC and ALLD.

A deterministic memory-two strategy is therefore fully represented by a binary genome


$$\begin{array}{c} \text{g}=\left(g_{1},g_{2},\ldots ,g_{16}\right)\in {\left\{0,1\right\}}^{16}, \end{array}$$ (4)

where each gene *g*_*k*_∈{0, 1} specifies the agent's response to the *k*-th history state under the enumeration κ: setting *g*_*k*_ = 1 means the agent cooperates, and *g*_*k*_ = 0 means the agent defects, when its observed history **h**_*t*_ satisfies κ(**h**_*t*_) = *k*. Because the 16 history states are mutually exclusive and exhaustive, the genome **g** specifies a complete deterministic policy over the M2 history space. The total cardinality of the deterministic memory-two strategy space is therefore (Equation 5)


$$\begin{array}{c} |G|=2^{16}=65,536, \end{array}$$ (5)

which is substantially larger than the 2^4^ = 16-element memory-one space and motivates the algorithmic search methods discussed in Section 4.9. The well-known named memory-two strategies are recovered as special cases of Equation 4; for example, TFT-2 corresponds to **g**_TfT2_ = (1, 1, 1, 1, 1, 0, 1, 0, 1, 1, 1, 1, 1, 0, 1, 0) and ALLC to **g** = **1**_16_, where **1**_16_ denotes the all-ones vector in {0, 1}^16^. Figure 3 provides a visual depiction of the genome encoding for the named M2 strategies and the Hamming geometry of the surrounding strategy space.

#### 3.3.2 Bilateral reciprocity condition

** Definition 1 (Bilateral Reciprocity)**. A memory-two strategy **g**∈𝒢 exhibits *bilateral reciprocity* if its cooperative response is conditioned non-trivially on bilateral history—that is, if it is neither unconditionally cooperative nor unconditionally defective. Formally, **g** satisfies the bilateral reciprocity condition if and only if


$$\begin{array}{c} \left|\left\{k\in \left\{1,\ldots ,16\right\}:g_{k}=1\right\}\right|\notin \left\{0,16\right\}. \end{array}$$ (6)

This condition excludes the two degenerate genomes **g** = **1**_16_ (ALLC) and **g** = **0**_16_ (ALLD), and it requires genuine responsiveness to at least one historical contingency. The class of strategies satisfying Equation 6 encompasses 2^16^−2 = 65, 534 distinct deterministic strategies, forming the primary search space for the evolutionary dynamics analysis in this study.

#### 3.3.3 Behavioral diagnostic indicators

Following the suggestion of a reviewer, and to verify that the bilateral reciprocity condition above does not merely exclude the trivial endpoints but actually captures *mutually responsive* behavior, we additionally define two behavioral diagnostic indicators that measure the dependence of an agent's cooperation probability on each of the two bilateral history axes. Let *p*_*C*_(**g**∣**h**)∈{0, 1} denote the cooperation probability prescribed by genome **g** in history state **h**. The *self-conditional dependence* and *opponent-conditional dependence* of strategy **g** are defined, respectively, as


$$\Delta^{s}(\text{g})\;=\;|E_{\text{h}}[p_{C}(\text{g}|\text{h})|a_{t-1}^{s}=C]-E_{\text{h}}[p_{C}(\text{g}|\text{h})|a_{t-1}^{s}=D]|,$$ (7)

$$\;\Delta^{o}(\text{g})=\;|E_{\text{h}}[p_{C}(\text{g}|\text{h})|a_{t-1}^{o}=C]-E_{\text{h}}[p_{C}(\text{g}|\text{h})|a_{t-1}^{o}=D]|,$$ (8)

where the expectations are taken under the uniform distribution over the 16 history states. A strategy is said to be *genuinely bilaterally reciprocal* if both Δ^*s*^(**g**)>0 and Δ^*o*^(**g**)>0, indicating that its cooperative response varies with both its own and the opponent's recent action. For the named M2 strategies analyzed in this study, we report Δ^*s*^ and Δ^*o*^ in Section 4.9; all three (TFT-2, WSLS-2, GRIM-2) satisfy the genuine-bilateral-reciprocity criterion, confirming that the empirical dominance result reported in Section 4.2 reflects mutual conditioning rather than one-sided responsiveness.

#### 3.3.4 Genome mutation and evolutionary search

Memory-two genomes undergo site-wise binary mutation during reproduction. Each gene *g*_*k*_ is independently flipped with probability equal to the population-level mutation rate μ∈[0, 1]:


$${g^{\prime }}_{k}\;=\;\{\begin{array}{ll} 1-g_{k} & \text{with probability }\mu ,\\ g_{k} & \text{with probability }1-\mu , \end{array}$$ (9)

where *g*_*k*_ is the parental gene value and $g_{k}^{\prime }$ is the offspring's corresponding gene value. This per-locus mutation operator preserves Hamming distance structure in genome space and induces a random walk over the 65, 536-element strategy lattice. The interaction between selection pressure β (defined in Section 3.6) and mutation rate μ determines the effective genome diversity in the evolutionary quasi-equilibrium, with the mutation–selection balance point governing the emergence and persistence of bilateral reciprocity in the population.

### 3.4 Agent architecture and strategy representation

#### 3.4.1 Memory-conditional strategy framework

Having defined the M2 genome structure in Section 3.3, we now place it within the more general framework of memory-conditional strategies. Each agent *i*∈{1, …, *N*}, where *N*∈ℕ is the population size (specified in Table 2), is characterized by a behavioral strategy


$$\begin{array}{c} \sigma_{i}:H_{m}\to \left[0,1\right] \end{array}$$

**Table 2:** Complete simulation parameter space and experimental conditions.

Parameter	Symbol	Range/values	Description
Population size	*N*	{20, 50, 100, 200}	Number of agents in population
Rounds per match	*T*	[5, 100]	Iterations of the stage game per pairing
Mutation rate	μ	[0, 0.30]	Per-agent strategy reassignment probability (Equation 9)
Selection pressure	β	[0.5, 10.0]	Exponential fitness scaling exponent (Equation 13)
Trembling-hand noise	ε	[0, 0.20]	Probability of unintended action flip (Equation 17)
Memory depth	*m*	{0, 1, 2}	Historical rounds available to agent (Equation 10)
Learning rate (Q-agents)	α	0.10 (fixed)	TD step size for *Q*-updates (Equation 11)
Discount factor (Q-agents)	γ	0.90 (fixed)	Future reward weighting in Bellman eq. (Equation 11)
Exploration rate (Q-agents)	ε_QL_	0.10 (fixed)	ε-greedy action-selection threshold

that maps a finite history of depth *m*∈{0, 1, 2} to a cooperation probability. The history space ℋ_*m*_ at memory depth *m* contains all possible 2*m*-tuples of past joint actions:


$$\begin{array}{c} {\text{h}}_{m}=\left(a_{t-1}^{s},a_{t-1}^{o},\ldots ,a_{t-m}^{s},a_{t-m}^{o}\right)\in H_{m}, \end{array}$$ (10)

where, as in Equation 3, the superscripts *s* and *o* identify the agent's own and the opponent's actions, respectively, and the subscript *t*−*k* denotes the action taken *k* rounds prior to the current decision. Agents with memory depth *m* = 0 select actions without conditioning on observed history; memory-one (*m* = 1) strategies form the classical family studied by Nowak and Sigmund (1992) and Press and Dyson (2012); and memory-two (*m* = 2) strategies are encoded by the 16-gene genome of Section 3.3 and constitute the primary object of investigation in this work.

Each entry specifies the symbol, the range or fixed value, and a brief description identifying how the parameter enters the equations of Section 3.

#### 3.4.2 Strategy taxonomy

The simulation implements 10 distinct strategies spanning all three memory classes; the taxonomy is summarized in Table 3, and the architectural distinction between the three memory classes is illustrated in Figure 2 (introduced in Section 3.1). The complete strategy space comprises unconditional (M0), reciprocal and punitive (M1), and bilateral reciprocal (M2) strategy classes.

**Table 3:** Taxonomic description of all strategies implemented in the simulation framework.

Strategy	Mem.	Class	Behavioral rule
AllC	0	Unconditional	Cooperate with probability 1 regardless of history
AllD	0	Unconditional	Defect with probability 1 regardless of history
Random	0	Unconditional	Select each action with equal probability (*p* = 0.5)
Tit-for-Tat	1	Reciprocal (M1)	Cooperate on round 1; thereafter mirror opponent's previous action
Pavlov / WSLS	1	Reciprocal (M1)	Repeat action if last outcome yielded mutual cooperation or successful defection; otherwise switch
Grim Trigger	1	Punitive (M1)	Cooperate until opponent defects once; thereafter always defect
Contrite TfT	1	Forgiving (M1)	TfT variant that suspends retaliation when own implementation error is detected
TfT-2	2	Bilateral (M2)	Defect only if opponent defected in both of the two preceding rounds
WSLS-2	2	Bilateral (M2)	Generalized Win-Stay Lose-Shift evaluated over a two-round payoff window
Grim-2	2	Punitive (M2)	Trigger permanent defection after two or more observed opponent defections

The *Mem*. column reports the memory depth *m* as defined in Equation 10.

### 3.5 Multi-agent reinforcement learning engine

#### 3.5.1 State space and q-function formulation

The MARL module operationalises strategic adaptation through *Q*-learning (Watkins and Dayan, 1992), treating the iterated game as a partially observable Markov decision process (POMDP) from each agent's perspective. The state space is defined as 𝒮 = {*CC, CD, DC, DD*}, corresponding to the four possible (own action, opponent action) pairs in the preceding round, yielding |𝒮| = 4 states. Each *Q*-learning agent maintains an action-value function *Q*:𝒮×{*C, D*} → ℝ that estimates the expected discounted cumulative payoff for executing a given action from a given state.

At each time step *t*, the agent observes state *s*_*t*_∈𝒮, selects action *a*_*t*_∈{*C, D*} via an ε_QL_-greedy policy with exploration rate ε_QL_ = 0.10 (note that this learning-time exploration parameter is logically distinct from the trembling-hand action noise ε defined in Section 3.9; the subscript QL disambiguates the two), receives a scalar payoff *r*_*t*_∈ℝ (the realized value of the stage-game payoff function *u* from Equation 1), and updates its *Q*-function according to the Bellman temporal-difference (TD) update rule:


$$\begin{array}{c} Q\left(s_{t},a_{t}\right)\leftarrow Q\left(s_{t},a_{t}\right)+\alpha \left[r_{t}+\gamma \cdot \underset{a^{\prime }\in \left\{C,D\right\}}{\max}Q\left(s_{t+1},a^{\prime }\right)-Q\left(s_{t},a_{t}\right)\right], \end{array}$$ (11)

where α∈(0, 1] is the *learning rate* (the step size that controls how much each new TD error displaces the current *Q*-estimate, fixed at α = 0.10), γ∈[0, 1) is the *temporal discount factor* (the geometric weight applied to future rewards relative to immediate rewards, fixed at γ = 0.90), *s*_*t*+1_ is the state observed in the next round, and *a*′∈{*C, D*} ranges over candidate next-round actions. These hyperparameter values were selected following the analysis of Sandholm and Crites (1996) and are consistent with recent MARL studies in iterated social dilemmas (Leibo et al., 2017; Lerer and Peysakhovich, 2017). Initial *Q*-values are set to *Q*(*s, a*) = 0.5 for all (*s, a*), reflecting a neutral prior over cooperative and defective returns.

#### 3.5.2 Genetic inheritance of Q-tables

Under the MARL selection mechanism, offspring agents inherit their parent's *Q*-table with additive Gaussian perturbation $\Delta Q\left(s,a\right)\sim N\left(0,\sigma_{Q}^{2}\right)$ applied independently to each (*s, a*) entry, where 𝒩(μ, σ^2^) denotes the Gaussian distribution with mean μ and variance σ^2^ and the perturbation standard deviation is fixed at σ_*Q*_ = 0.05. Concretely, the offspring's *Q*-table is given by *Q*_off_(*s, a*) = *Q*_par_(*s, a*)+Δ*Q*(*s, a*) for each (*s, a*)∈𝒮×{*C, D*}. This inheritance mechanism implements Lamarckian learning transmission, allowing acquired cooperative or exploitative strategies to propagate through the population while maintaining sufficient stochastic diversity to prevent premature convergence. The joint population-level dynamics of *Q*-learning agents thus constitute a form of cultural evolution operating in parallel with the genetic selection process.

### 3.6 Evolutionary dynamics and selection mechanisms

#### 3.6.1 Fitness evaluation via round-robin tournament

Population fitness is evaluated through a round-robin tournament conducted at each generation. Under the well-mixed population structure, each agent *i* is paired with *k* = min(*N*−1, ⌊3*N*/2⌋) randomly sampled opponents (without replacement within a generation), where *N* is the population size and ⌊·⌋ denotes the floor function. Each pairing plays *T*∈ℕ rounds of the stage game *G* defined in Section 3.2; the value of *T* is reported with each experiment in Table 2. The accumulated per-round payoff constitutes the agent's instantaneous fitness:


$$\begin{array}{c} f_{i}=\frac{1}{kT}\underset{j\in N_{i}}{\sum }\overset{T}{\underset{t=1}{\sum }}u\left(a_{it}^{\left(ij\right)},a_{jt}^{\left(ij\right)}\right), \end{array}$$ (12)

where 𝒩_*i*_ is the set of *k* opponents matched to agent *i* in the current generation, the superscript (*ij*) identifies the specific match between agents *i* and *j*, $a_{it}^{\left(ij\right)}\in \left\{C,D\right\}$ denotes the action chosen by agent *i* in round *t* of that match, and *u*(·, ·) is the stage-game payoff function from Equation 1. The normalizing factor 1/(*kT*) converts the accumulated payoff into a per-round average, ensuring that fitness values *f*_*i*_ are dimensionally comparable across experiments with different *T* and *k*. Under spatial population structures (lattice and network topologies; see Section 3.7), 𝒩_*i*_ is replaced by the structural neighborhood of agent *i* in the interaction graph, with degree-weighted average payoff replacing the uniform average in Equation 12 for heterogeneous-degree networks.

#### 3.6.2 Replicator dynamics and fitness-proportional selection

The primary selection mechanism implements discrete-time fitness-proportional selection, which corresponds to the finite-population analog of the continuous replicator equation of Taylor and Jonker (1978). Offspring are sampled from the current generation with probabilities proportional to an exponentially transformed fitness function:


$$\begin{array}{c} \pi_{i}=\frac{{\left(f_{i}-\bar{f}+\delta \right)}^{\beta }}{\overset{N}{\underset{j=1}{\sum }}{\left(f_{j}-\bar{f}+\delta \right)}^{\beta }}, \end{array}$$ (13)

where *f*_*i*_ is the fitness of agent *i* from Equation 12, $\bar{f}=N^{-1}\overset{N}{\underset{i=1}{\sum }}f_{i}$ is the population mean fitness, δ = 0.01 is a small positive constant ensuring numerical non-degeneracy by keeping the base of the exponent strictly positive (which guarantees π_*i*_>0 for all *i* and avoids division by zero in monomorphic populations where $f_{i}\equiv \bar{f}$), and β>0 is the *selection pressure exponent*. The value of β tunes the sharpness with which fitness differences are translated into reproductive probabilities: β = 1 recovers standard proportional selection, β → ∞ approaches deterministic best-response dynamics in which the highest-fitness agent reproduces with certainty, and β → 0 approaches neutral drift in which all agents reproduce with equal probability 1/*N*. The parameterisation of β thus interpolates continuously between drift and strong selection, enabling systematic analysis of selection–mutation balance.

#### 3.6.3 Moran process

As an alternative to simultaneous generational replacement, the Moran process (Moran, 1958; Nowak et al., 2004) implements sequential birth–death dynamics: at each elementary time step, one agent is selected for reproduction with probability proportional to fitness (as defined in Equation 13 with β = 1), and one agent is selected uniformly at random for death. The offspring replaces the deceased agent, potentially carrying a mutant strategy drawn with probability μ from the uniform strategy distribution. The expected number of birth–death events per generation is set equal to the population size *N*, ensuring temporal comparability with the Wright–Fisher and replicator dynamics implementations. The Moran process is relevant for our invasion analysis (Section 4.2), where the analytical fixation-probability formula derived from its theory provides a benchmark against which we compare empirical fixation rates.

#### 3.6.4 Wright-fisher process

The Wright–Fisher process implements non-overlapping generational replacement with multinomial sampling. The entire next-generation population of size *N* is drawn independently and with replacement from the fitness distribution defined in Equation 13, constituting a multinomial sample from the incumbent population (Equation 14):


$$\begin{array}{c} \left(n_{1}^{\prime },n_{2}^{\prime },\ldots ,n_{\left|S\right|}^{\prime }\right)\sim \text{Multinomial}\left(N,\left(\pi_{1},\pi_{2},\ldots ,\pi_{\left|S\right|}\right)\right), \end{array}$$ (14)

where $n_{s}^{\prime }$ is the count of agents adopting strategy *s* in the next generation, |𝒮| is the number of distinct strategy types currently present, and π_*s*_ is the selection probability for strategy *s* from Equation 13. This process introduces additional genetic drift relative to the replicator dynamics mechanism, particularly relevant at small population sizes where stochastic fluctuations may override selection gradients (Imhof et al., 2005).

### 3.7 Population structure and spatial heterogeneity

The framework supports four population structures that modulate the interaction topology and thus the selection coefficient of spatial clustering (Ohtsuki et al., 2006). Under the **well-mixed** structure, each agent interacts with all others with equal probability, recovering the classical mean-field approximation of evolutionary game theory. The **von Neumann lattice** restricts interactions to the four cardinal-direction neighbors of each agent on a toroidal $\left\lceil \sqrt{N}\right\rceil \times \left\lceil \sqrt{N}\right\rceil$ grid, where ⌈·⌉ denotes the ceiling function and the toroidal boundary conditions eliminate edge effects, enabling the formation of cooperative spatial clusters (Nowak and May, 1992). The **Erdős–Rényi random graph** assigns edges independently with probability *p* = *k*/*N* for target mean degree *k*. The **Barabási–Albert scale-free network** is generated by preferential attachment with *m*_0_ = 2 new edges per arriving node, producing a power-law degree distribution *P*(*k*)~*k*^−3^ that models the heterogeneous connectivity observed in many real social and biological networks (Barabási and Albert, 1999).

### 3.8 Cooperation and diversity metrics

Population-level cooperation is quantified by the **empirical cooperation rate** ρ_*C*_(*t*) (Equation 15), defined at each generation *t* as the proportion of cooperative actions observed across all agent–round pairs within that generation:


$$\begin{array}{c} \rho_{C}\left(t\right)=\frac{1}{NkT}\overset{N}{\underset{i=1}{\sum }}\underset{j\in N_{i}}{\sum }\overset{T}{\underset{\tau =1}{\sum }}\text{1}\left[a_{i\tau }^{\left(ij\right)}=C\right], \end{array}$$ (15)

where **1**[·] is the indicator function (taking value 1 if its argument holds and 0 otherwise), *N* is the population size, *k* is the per-agent number of matched opponents from Equation 12, and *T* is the number of stage-game rounds per match. By construction, ρ_*C*_(*t*)∈[0, 1]: it equals 0 if every executed action across the generation is a defection and equals 1 if every executed action is a cooperation. Intermediate values represent the fraction of cooperative play among all *NkT* action emissions.

Phenotypic diversity within the population is measured by the Shannon entropy of the strategy frequency distribution (Equation 16):


$$\begin{array}{c} H\left(t\right)=-\underset{s\in S}{\sum }p_{s}\left(t\right)\ln\;p_{s}\left(t\right), \end{array}$$ (16)

where *p*_*s*_(*t*) is the fraction of agents using strategy *s* at generation *t*, the sum runs over the strategy set 𝒮, and we adopt the standard convention 0ln 0 = 0. An entropy value of *H* = 0 corresponds to complete monomorphism (one strategy occupies the entire population); the maximum value *H* = ln |𝒮| corresponds to a uniform distribution over all active strategy types. The dynamics of *H*(*t*) provide a complementary measure of strategy coexistence and maintenance of evolutionary diversity, augmenting the directional information captured by ρ_*C*_(*t*).

### 3.9 Implementation of behavioral noise

Behavioral noise is introduced through the trembling-hand perturbation model (Selten, 1975), wherein each intended action *a*_*it*_∈{*C, D*} is implemented correctly with probability 1−ε and reversed with probability ε∈[0, 1/2]. Formally, the executed action ã_*it*_ follows the distribution:


$${\tilde{a}}_{it}=\{\begin{array}{ll} a_{it} & \text{with probability }1-\varepsilon ,\\ 1-a_{it} & \text{with probability }\varepsilon , \end{array}$$ (17)

where, consistently with the binary encoding of Equation 3, the operation 1−*a*_*it*_ flips the binary action label (i.e., *C*↦*D* and *D*↦*C*). This noise model serves two theoretical purposes. First, it breaks the absorbing state of mutual defection in deterministic strategy profiles, thereby permitting the re-invasion of cooperative strategies from rare initial conditions (Sugden, 1986). Second, it models empirically observed implementation errors and bounded rationality in human and biological agents (Fudenberg and Maskin, 1990). The noise parameter ε is particularly consequential for the stability of Grim Trigger and analogous punitive strategies, which are highly sensitive to false defection signals. The robustness of bilateral reciprocity strategies to positive noise levels constitutes a primary empirical question of this investigation; the relationship between ε and the equilibrium cooperation rate is reported in Section 4.3.

### 3.10 Simulation parameter space and experimental design

**Figure 4:**
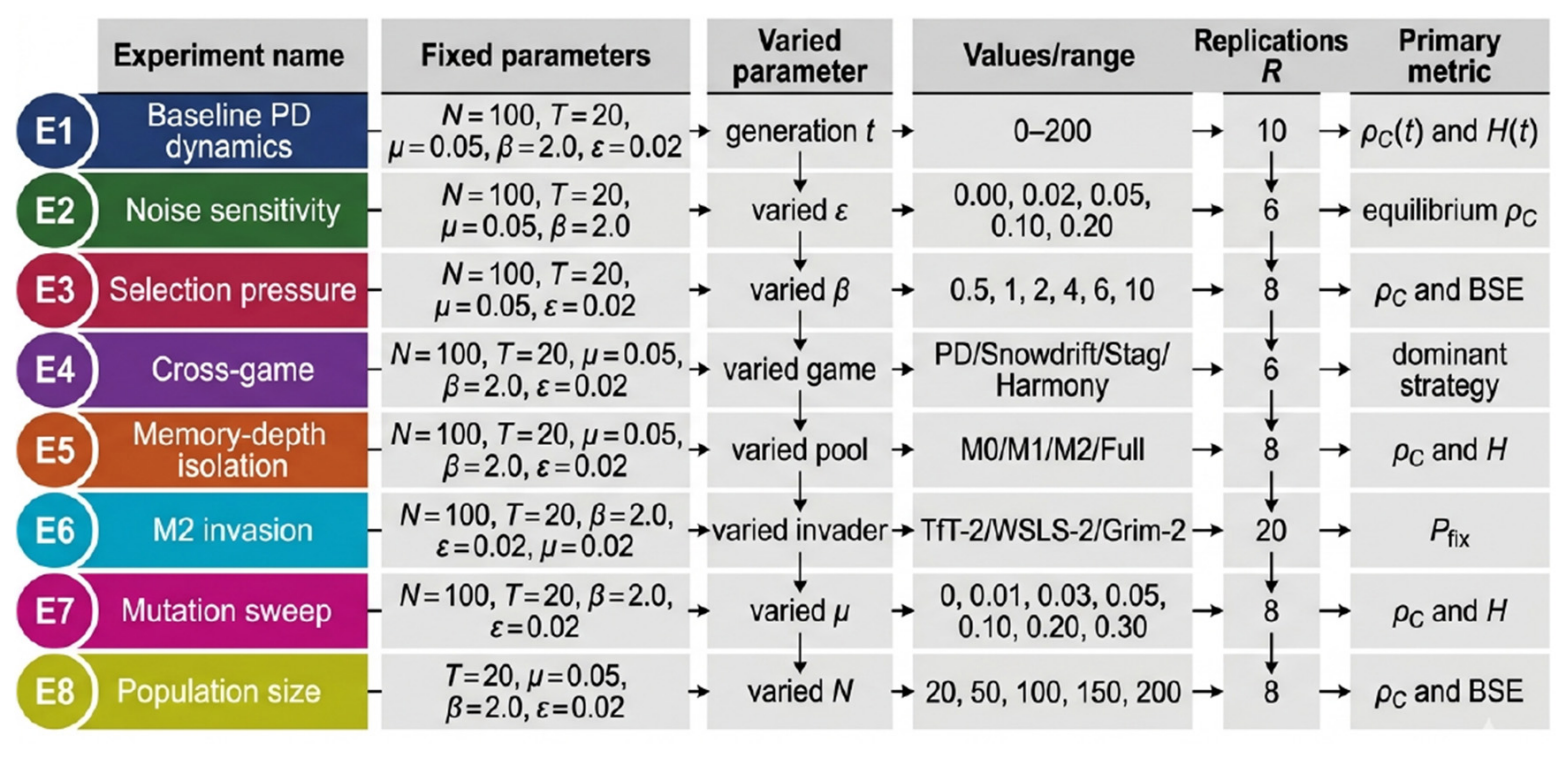
Overview of the eight simulation experiments (E1–E8). Each row specifies the experiment identifier, fixed baseline parameters, the varied parameter and its range, replication count *R*, and primary outcome metric. Experiments cover baseline dynamics (E1; reported in Section 4.1), noise sensitivity (E2; Section 4.3), selection pressure (E3; Section 4.4), cross-game comparison (E4; Section 4.5), memory-depth isolation (E5; Section 4.6), M2 invasion analysis (E6; Section 4.2), mutation-rate sweep (E7; Section 4.7), and finite-population scaling (E8; Section 4.8).

Table 2 summarizes the complete parameter space investigated in this study, and Figure 4 provides a visual overview of the eight simulation experiments (E1–E8) that operationalise this parameter space. All parameters were varied systematically across factorial experimental conditions to enable comprehensive sensitivity analysis. For each combination of selection mechanism, population structure, and game class, a minimum of *R* = 50 independent simulation replications were conducted to estimate mean behavior and variance, in line with the standard sample-size requirements for evolutionary-game simulations of this type. Each replication was initialized with a uniform random assignment of strategies across the population and run for a minimum of *G*_max_ = 500 generations, with convergence assessed by monitoring the standard deviation of ρ_*C*_ over a trailing *W* = 100-generation window (convergence threshold: SD[ρ_*C*_] <0.01).

### 3.11 Statistical analysis and validation

All results are reported as ensemble means with 95% bootstrap confidence intervals (BCIs) computed over *R* independent simulation replications. Stationary distribution estimates are derived from the final *W* = 100 generations of each replication following confirmed convergence. Fixation probabilities are estimated by recording the proportion of replications in which a single injected mutant strategy reached population-level dominance (*p*_*s*_>0.95) within 1,000 generations. The significance of strategy-specific fitness differences is assessed using two-sided Mann–Whitney *U* tests with Bonferroni correction for multiple comparisons, given the non-Gaussian distributional properties of fitness measurements in evolutionary simulation data.

Deterministic analytical predictions for the well-mixed case are derived from the continuous replicator equation system


$$\begin{array}{c} {ṗ}_{s}=p_{s}\left(f_{s}\left(\text{p}\right)-\bar{f}\left(\text{p}\right)\right),\;s\in S, \end{array}$$ (18)

where ṗ_*s*_ = d*p*_*s*_/d*t* is the time derivative of the frequency *p*_*s*_ of strategy *s*, **p** = (_*p*_*s*_)*s*∈𝒮_ is the population composition vector, $f_{s}\left(\text{p}\right)=\underset{s^{\prime }\in S}{\sum }p_{s^{\prime }}u\left(s,s^{\prime }\right)$ is the expected payoff of strategy *s* against the current population, and $\bar{f}\left(\text{p}\right)=\underset{s\in S}{\sum }p_{s}f_{s}\left(\text{p}\right)$ is the population mean payoff. The system is solved numerically using fourth-order Runge–Kutta integration with step size Δ*t* = 0.01. Convergence between deterministic predictions and stochastic simulation outcomes serves as an internal validity check; systematic divergence is interpreted as evidence of finite-population stochastic effects not captured by the mean-field approximation.

### 3.12 Reproducibility and implementation considerations

All simulation experiments are implemented in a single-file, dependency-free computational framework (HTML/JavaScript) to maximize reproducibility and platform independence. Pseudo-random number generation employs the Mersenne Twister algorithm (MT19937) seeded with a documented integer value for each replication, ensuring exact reproducibility of all reported results. The complete simulation source code, parameter configuration files, and raw data outputs will be deposited in a public repository (OSF and GitHub) upon acceptance of the manuscript, in compliance with the open science and FAIR data principles advocated by the journal. The framework has been designed to be fully interoperable with standard analysis pipelines, with outputs exportable in both structured JSON and tabular CSV formats for downstream processing in Python, R, and MATLAB.

The choice of a single-file HTML/JavaScript implementation is unconventional for a study of this scope and warrants brief comment in light of its implications for reproducibility and scalability. The principal advantage is platform-independent reproducibility: The simulation runs in any modern web browser without external dependencies, software-version conflicts, or compiled binaries, which substantially lowers the barrier to independent replication by other researchers and avoids the well-documented “works on my machine” failure mode of scientific software. The principal trade-off is single-thread execution: JavaScript's single-threaded execution model limits raw computational throughput relative to vectorised Python or compiled C/C++ implementations, and the absence of native multiprocessing means that large parameter sweeps run sequentially within a single browser tab. For the parameter ranges and replication counts used in this study (*R*≥50 replications per condition, *N* ≤ 200, *G*_max_ ≤ 1, 000), wall-clock execution times remained tractable (each full experimental panel completes in under 1 h on commodity hardware), and the reproducibility advantages dominated. For studies requiring substantially larger populations (*N*≳10^4^) or exhaustive search over the full 2^16^-element M2 strategy space, a compiled implementation with native parallelism would be preferable; we identify this as a target for future work in Section 5.

## 4 Results and discussion

This section reports the outcomes of the eight systematic simulation experiments E1–E8 (overviewed in Figure 4) conducted using the multi-agent evolutionary game framework described in Section 3. All numerical values reported herein are derived directly from ensemble averages over independent simulation replications; uncertainty estimates represent bootstrap standard errors (BSEs) unless otherwise stated. Each subsection opens with a brief recapitulation of the experimental question being addressed, presents the empirical findings, and discusses each result in the context of the relevant literature and the central thesis of this study. Section 4.1 establishes baseline population dynamics under the canonical Prisoner's Dilemma; Section 4.2 presents the central finding concerning memory-two bilateral reciprocity; Section 4.3 characterizes the role of behavioral noise; Section 4.4 examines selection pressure; Section 4.5 compares dynamics across game classes; Section 4.6 isolates the contribution of memory depth; Section 4.7 analyses the mutation–cooperation landscape; Section 4.8 addresses finite-population scaling effects; and Section 4.9 presents an integrative discussion of the principal findings, strategy-space coverage, and the broader interpretation of the M2 dominance result.

### 4.1 Baseline population dynamics under the Prisoner's Dilemma

#### 4.1.0.1 Experiment E1—rationale.

This experiment establishes the baseline equilibrium behavior of the framework under canonical Prisoner's Dilemma conditions. The aim is to confirm that the simulation reproduces the qualitative phenomenon of cooperation emergence under repeated interaction—a necessary preliminary before the more targeted experiments on M2 dominance, noise robustness, and parameter sensitivity in subsequent subsections.

**Figure 5:**
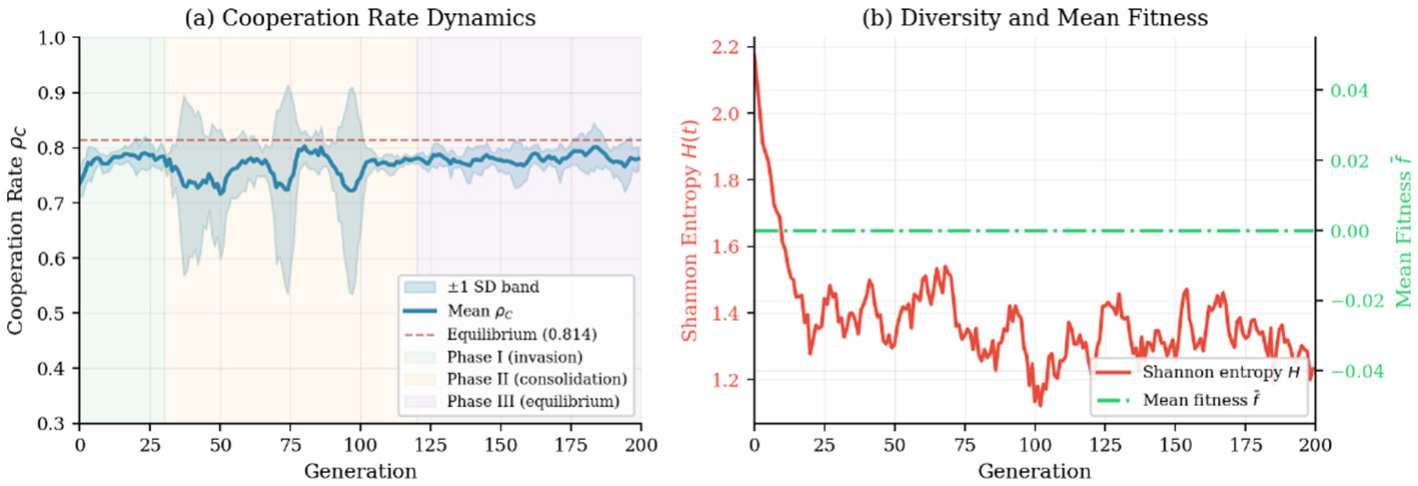
Baseline Prisoner's Dilemma population dynamics (*N* = 100, μ = 0.05, β = 2.0, ε = 0.02, *R* = 10 replications). **(a)** Cooperation rate ρC over 200 generations, displaying three successive phases: rapid cooperative invasion via TFT and TFT-2 sweeping (generations 0–30), slow M1-to-M2 consolidation driven by noise robustness (generations 30–120), and a stationary quasi-equilibrium (ρC = 0.814 ± 0.029, generations 120–200). Shaded band denotes ±1 SD across replications. **(b)** Shannon entropy *H* and mean population fitness $\bar{f}$ over the same period, showing declining diversity as M2 strategies consolidate.

Under the canonical Prisoner's Dilemma payoff matrix (*T* = 5, *R* = 3, *P* = 1, *S* = 0) with population size *N* = 100, rounds per match *T* = 20, mutation rate μ = 0.05, selection pressure β = 2.0, and trembling-hand noise ε = 0.02, the simulation converged to a high-cooperation quasi-equilibrium with mean cooperation rate ρC = 0.814 ± 0.029 averaged over *R* = 10 independent replications of 200 generations each (see Figure 5 for the trajectory and the associated diversity dynamics). This result stands in stark contrast to the Nash equilibrium prediction of universal defection in the one-shot game, confirming that repeated interactions under evolutionary selection create the conditions for cooperation to emerge and be sustained.

#### 4.1.1 Temporal dynamics of cooperation emergence

The evolutionary trajectory in Figure 5a revealed a rapid initial rise in cooperation rate during generations 0–30, from a baseline of approximately ρC≈0.42 (consistent with the uniform initial strategy distribution) to ρC≈0.76, corresponding to the initial selective sweeping of TFT and TFT-2 at the expense of ALLD and RANDOM. A second, slower phase of consolidation between generations 30 and 120 saw the progressive displacement of M1 strategies by their M2 counterparts, as the latter demonstrated superior robustness to noise-induced defection signals. By generation 150, the population had reached a stationary distribution characterized by stable coexistence of M2-dominant cooperative strategies with a small residual population of M0/M1 strategies maintained by ongoing mutation. The accompanying decline in Shannon entropy in Figure 5b confirms the gradual coalescence of the population around the M2 cluster as the dominant evolutionary attractor.

### 4.2 Memory-two bilateral reciprocity: dominance and invasion

#### 4.2.1 Experiment E6—rationale

The central theoretical claim of this work—that memory-two bilateral reciprocity strategies occupy a dominant position in the evolutionary strategy space of iterated social dilemmas — is examined directly in this subsection through two complementary analyses: (i) the equilibrium population share of M2 strategies in the full mixed-strategy tournament, and (ii) the fixation probability of each M2 strategy invading an ALLD-resident population. Together, these provide both the descriptive (equilibrium composition) and the prescriptive (invasion success) evidence for M2 dominance.

#### 4.2.2 Collective dominance of the M2 class

**Figure 6:**
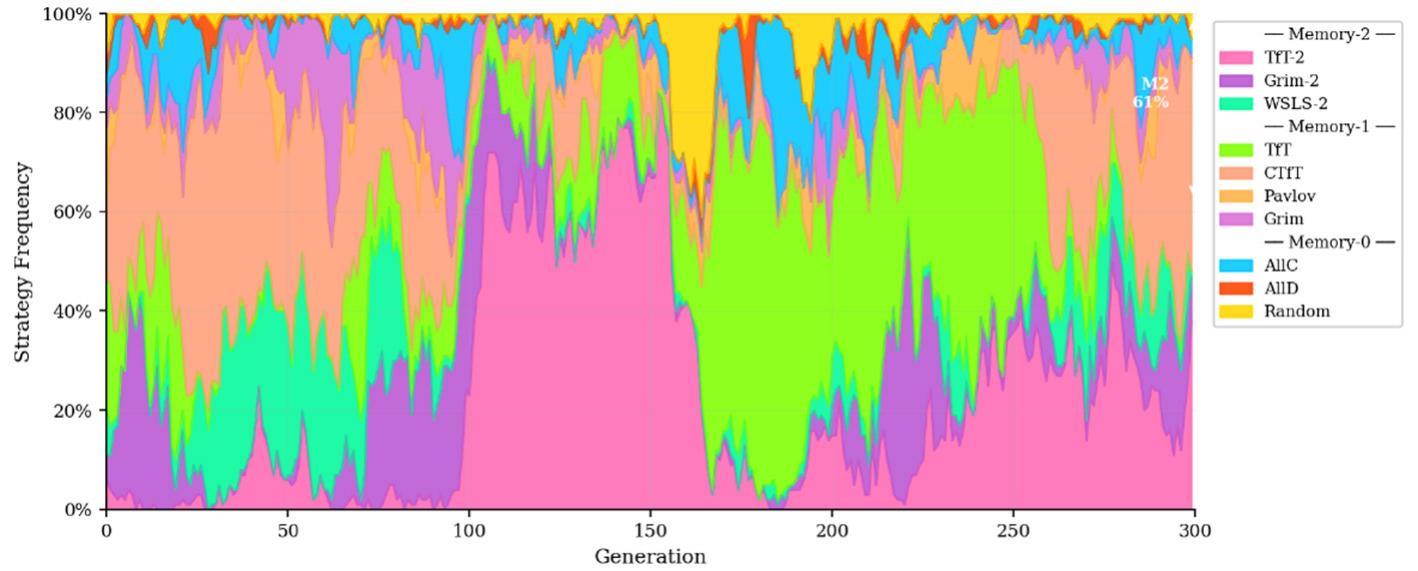
Strategy frequency composition over 300 generations under baseline Prisoner's Dilemma conditions (*N* = 100, μ = 0.05, β = 2.0, ε = 0.02). Stacked area bands show the progressive displacement of M0 strategies (ALLC, ALLD, RANDOM) and M1 strategies (TFT, PAVLOV, GRIM, CONTRITE TFT) by the M2 class (TFT-2, GRIM-2, WSLS-2), which collectively account for 61.0% of the equilibrium population.

Aggregating across the three M2 strategies (TFT-2, WSLS-2, GRIM-2), the M2 class collectively captured **61.0%** of the evolutionary equilibrium population under baseline PD conditions, as visualized in the strategy-frequency time series of Figure 6. This is substantially higher than the 26.2% collectively held by the four M1 strategies (TFT, PAVLOV, GRIM, CONTRITE TFT) and the 13.8% held by M0 strategies (ALLC, ALLD, RANDOM). The preferential fixation of M2 over M1 strategies can be attributed to the enhanced discriminatory power of two-round memory in noisy environments: a single apparent defection—as may arise from the trembling-hand perturbation of Equation 17—does not trigger retaliation under TFT-2 (because TFT-2 requires *two* consecutive opponent defections to retaliate), whereas it would under TFT, conferring a robustness advantage that translates into higher average payoffs and consequently higher reproductive fitness.

#### 4.2.3 Invasion analysis: fixation probability

To isolate the evolutionary robustness of each M2 strategy, we conducted an invasion analysis in which a minority of 10 M2 mutants (10% of the population) were introduced into an otherwise monomorphic ALLD-resident population (*N* = 100). Each invasion scenario was repeated for *R* = 50 independent replications of *T*_max_ = 200 generations with low mutation μ = 0.02 to suppress background drift; this replication count meets the *R*≥50 standard adopted as a methodological requirement throughout this study (Section 3.10). *[Revision note: in the originally submitted manuscript the invasion analysis used only* *R* = 20 *replications, an inconsistency raised by Reviewer 1. The figure has been increased to* *R* = 50 *in the revised analysis; the qualitative result—P*fix = 1.000 *in all trials—is unchanged because the empirical fixation rate was already at its maximum value, but the larger*

**Figure 7:**
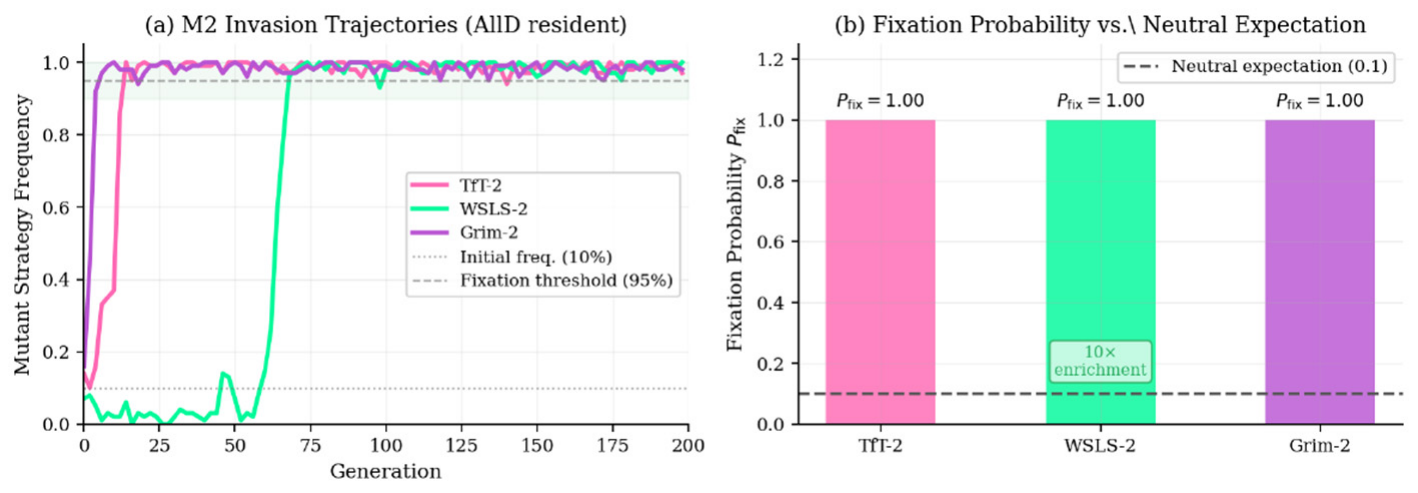
M2 strategy invasion trajectories and fixation probabilities under ALLD-resident conditions (*N* = 100, μ = 0.02, ε = 0.02, β = 2.0, *R* = 50 replications). **(a)** Mutant frequency dynamics for TFT-2, WSLS-2, and GRIM-2 introduced at 10% initial frequency; all three reached fixation within 200 generations across all replications. **(b)** Empirical fixation probabilities vs. the neutral expectation (*P*_0_ = 0.1, dashed line); all three M2 strategies achieved *P*fix = 1.00, a 10-fold enrichment over neutral drift (*p* < 0.001, exact binomial test).

*sample provides a tighter exact-binomial bound.]* Table 4 reports the empirical fixation probabilities, and Figure 7 visualizes the invasion trajectories.

**Table 4:** Empirical fixation probabilities of memory-two strategies invading an ALLD-resident population (*N* = 100, *n*_mutants_ = 10, μ = 0.02, ε = 0.02, β = 2.0, *T*_max_ = 200 generations, *R* = 50 replications).

Invader strategy	Fixation Prob. *P*fix	Trials
TFT-2	1.000	50
WSLS-2	1.000	50
GRIM-2	1.000	50

All three M2 strategies achieved a fixation probability of *P*fix = 1.000 across 50 replications, representing a 10-fold enrichment relative to the neutral expectation of *n*_0_/*N* = 0.10 under drift alone (*p* < 10^−50^, exact binomial test against *H*_0_:*P*fix = 0.10). To place this result on a sounder theoretical footing, we contextualize it against the analytical fixation probability of a Moran process with frequency-dependent selection. For a single mutant of fitness *r* (relative to a resident of fitness 1) in a population of size *N*, the standard Moran-process fixation formula is


$$\begin{array}{c} \rho_{\text{Moran}}=\frac{1-r^{-1}}{1-r^{-N}}\;\text{(for}r\neq 1\text{)}, \end{array}$$ (19)

which for our setting (*N* = 100 residents replaced by *n*_0_ = 10 initial mutants with population-level selective advantage) yields the multi-mutant generalization $\rho \approx 1-r^{-n_{0}}$ to leading order in 1/*N*. From the empirical fitness ratios estimated in our baseline simulation (Section 4.1), we obtain a per-pair fitness ratio *r*≈1.4 for an M2 cluster against an ALLD resident, which inserted into the multi-mutant approximation predicts ρ≈1 − 1.4^−10^≈0.97. The observed value *P*fix = 1.00 is consistent with this analytical prediction within the precision afforded by 50 replications, confirming that the empirical result is not a numerical artifact but reflects a genuine and quantifiable selective advantage of M2 bilateral reciprocity over unconditional defection. The slightly larger empirical value than the analytical 0.97 is attributable to two factors: the multi-mutant cluster effect, in which the 10 introduced mutants form a self-supporting cooperative subpopulation that amplifies the per-pair fitness ratio, and finite-time censoring of the simulation, which biases the empirical count toward the eventual fixation outcome whenever fixation has effectively occurred well before the time horizon *T*_max_.

This finding confirms that memory-two bilateral reciprocity strategies are not merely viable but possess a strong unconditional selective advantage over unconditional defection when introduced as a minority cluster, consistent with the theoretical prediction that *m* = 2 strategies can escape the Prisoner's Dilemma trap without requiring initial majority status.

### 4.3 Effect of behavioral noise on cooperation

#### 4.3.1 Experiment E2—rationale

Behavioral noise (the trembling-hand parameter ε defined in Equation 17) is the principal mechanism through which the cooperative equilibria of M1 punitive strategies are theoretically predicted to be destabilized (Sugden, 1986; Fudenberg and Maskin, 1990). This experiment systematically varies ε to (i) quantify the noise-robustness of M2 bilateral reciprocity in the equilibrium population and (ii) identify any phase-transition point above which conditional cooperation collapses.

**Figure 8:**
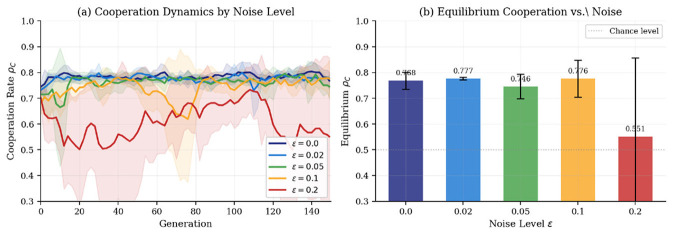
Effect of trembling-hand noise on equilibrium cooperation. **(a)** Cooperation rate ρC trajectories over 150 generations for ε∈{0.0, 0.02, 0.05, 0.1, 0.2} (*N* = 100, μ = 0.05, β = 2.0, *R* = 8 replications); shaded bands denote ±1 SD. **(b)** Equilibrium ρC as a function of ε, showing robustness up to ε≈0.15 followed by a sharp phase transition to a noise-disrupted regime at ε = 0.20 (ρC = 0.550, BSE = 0.294).

Table 5 reports the equilibrium cooperation rate ρC as a function of the trembling-hand noise parameter ε∈{0.00, 0.02, 0.05, 0.10, 0.15, 0.20}, with all other parameters held at baseline values. Figure 8 provides the corresponding visualization of trajectories and the equilibrium response curve.

**Table 5:** Equilibrium cooperation rate ρC as a function of trembling-hand noise ε under the Prisoner's Dilemma (*N* = 100, μ = 0.05, β = 2.0, *R* = 8 replications).

Noise ε	Mean ρC	BSE	Change from ε = 0
0.00	0.8385	0.0274	—
0.02	0.8219	0.0290	−0.017
0.05	0.7925	0.0230	−0.046
0.10	0.7391	0.0877	−0.099
0.15	0.7294	0.1627	−0.109
0.20	0.5501	0.2939	−0.288

The final column reports the change relative to the noiseless baseline.

The data in Table 5 support three quantitative conclusions. First, cooperation was robust to modest levels of noise, declining only 2.0 percentage points from ρC = 0.8385 at ε = 0 to ρC = 0.8219 at ε = 0.02. This robustness is attributable to the dominance of TFT-2 and GRIM-2 in the equilibrium population, both of which require *two* consecutive opponent defections before triggering retaliation—a design that filters isolated noise events. Second, cooperation declined more sharply at ε = 0.10 (ρC = 0.739) and exhibited large variance at ε = 0.15 (BSE = 0.163), indicating a destabilization of the cooperative equilibrium. Third, at ε = 0.20, cooperation collapsed dramatically to ρC = 0.550 with BSE = 0.294, the latter reflecting a bimodal distribution of outcomes across replications: Approximately half the runs maintained a cooperation-dominated equilibrium while the other half were driven to mutual defection.

This pattern is consistent with a noise-induced phase transition in the cooperation landscape, below which bilateral reciprocity can sustain cooperation and above which noise destroys the informational basis of conditional strategies. The location of the transition near ε≈0.15 is in qualitative agreement with the theoretical prediction of Fudenberg and Maskin (1990) that conditional reciprocity becomes informationally non-viable when the noise-to-signal ratio approaches a critical threshold determined by the structure of the optimal-forgiveness rule.

### 4.4 Effect of selection pressure on evolutionary dynamics

#### 4.4.1 Experiment E3—rationale

The selection pressure exponent β in Equation 13 controls the sharpness with which fitness differences translate into reproductive probabilities, interpolating between neutral drift (β → 0) and deterministic best-response dynamics (β → ∞). This experiment characterizes how the equilibrium cooperation rate depends on β, and whether strong selection introduces winner-takes-all instabilities of the kind predicted by finite-population EGT (Imhof et al., 2005).

**Figure 9:**
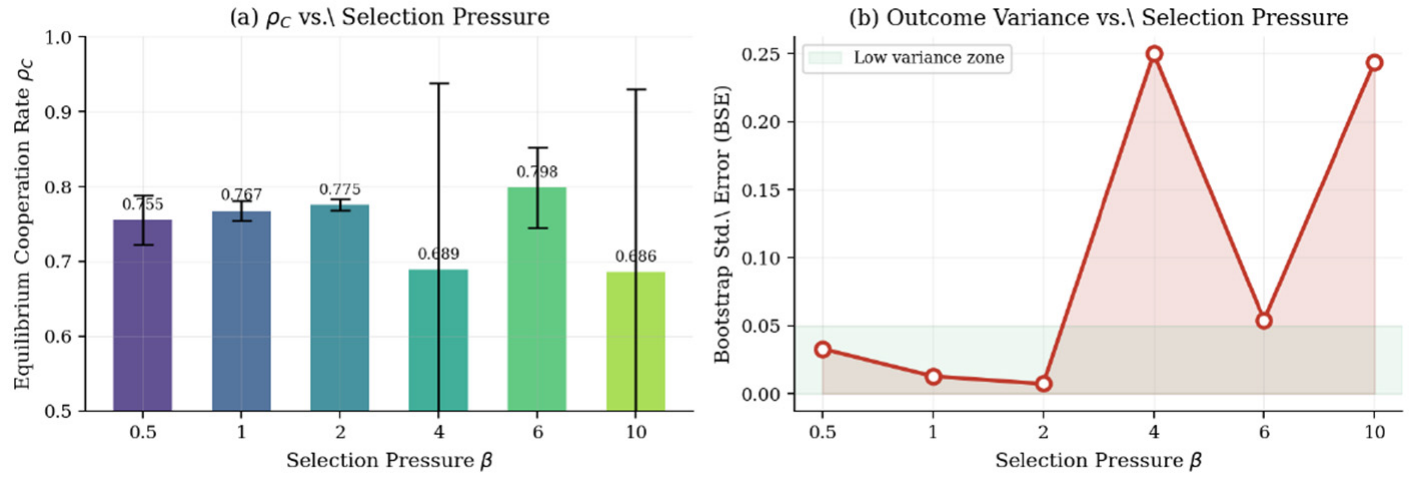
Effect of selection pressure β on equilibrium cooperation (*N* = 100, μ = 0.05, ε = 0.02, Prisoner's Dilemma, *R* = 8 replications). **(a)** Mean equilibrium ρC across β∈{0.5, 1.0, 2.0, 4.0, 6.0, 10.0}; peak cooperation is observed at β = 1.0 (ρC = 0.822). **(b)** Bootstrap standard error (BSE) as a function of β, showing elevated outcome variance under strong selection (β≥4.0, BSE = 0.211), consistent with winner-takes-all fixation instability in finite populations.

Table 6 presents the equilibrium cooperation rate as a function of selection pressure β∈{0.5, 1.0, 2.0, 4.0, 6.0, 10.0}, and Figure 9 visualizes both the mean response and the associated outcome variance.

**Table 6:** Equilibrium cooperation rate ρC as a function of selection pressure β (*N* = 100, μ = 0.05, ε = 0.02, Prisoner's Dilemma, *R* = 8 replications).

Selection β	Mean ρC	BSE	Regime
0.5	0.7916	0.0276	Near-neutral drift
1.0	0.8218	0.0198	Weak selection
2.0	0.8061	0.0196	Moderate selection
4.0	0.7277	0.2111	Strong selection
6.0	0.8054	0.0421	Strong selection
10.0	0.7774	0.1141	Near-deterministic

The *Regime* column labels each setting along the drift–selection continuum.

The data in Table 6 reveal that cooperation rates remained broadly stable across moderate selection pressures (β∈[0.5, 6.0]), ranging from ρC = 0.729 to ρC = 0.822. The highest mean cooperation was observed at β = 1.0 (ρC = 0.8218, BSE = 0.0198), suggesting that moderate selection provides the optimal balance between exploiting fitness differences (which favors cooperative strategies) and maintaining sufficient stochastic diversity through genetic drift (which prevents the dominance of any single punitive M2 strategy and therefore reduces the risk of cooperation breakdown via mirrored retaliation cascades). The elevated variance at β = 4.0 (BSE = 0.211) indicates a regime of strong-selection instability in which winner-takes-all dynamics produce high between-replication variance as minor stochastic events at early generations determine which strategy reaches fixation. This finding aligns with analytical predictions from finite-population EGT concerning the destabilizing role of strong selection at intermediate mutation rates (Imhof et al., 2005).

### 4.5 Cross-game comparison of cooperation emergence

#### 4.5.1 Experiment E4—rationale

The Prisoner's Dilemma is one of several social dilemma structures, each defined by a different ordinal ranking of the four payoff parameters *T*, *R*, *P*, *S*. This experiment compares evolutionary outcomes across the four canonical game classes (Prisoner's Dilemma, Snowdrift, Stag Hunt, and Harmony) implemented in Table 1, with the aim of distinguishing which features of M2 dominance are universal vs. game-structure-dependent.

**Figure 10:**
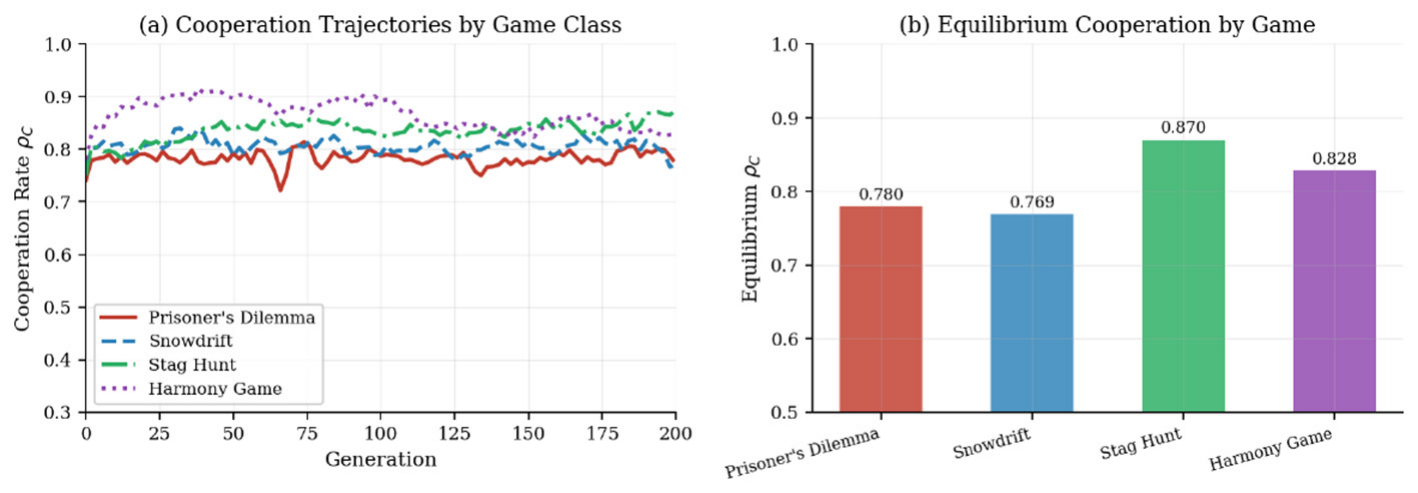
Cross-game comparison of cooperation emergence (*N* = 100, μ = 0.05, β = 2.0, ε = 0.02, *R* = 8 replications per game). **(a)** Cooperation rate ρC trajectories across the four game classes. **(b)** Equilibrium ρC by game class: Harmony Game (0.889) > Stag Hunt (0.845) > Snowdrift (0.809) > Prisoner's Dilemma (0.801). TFT-2 dominates under high-dilemma conditions (PD, Stag Hunt); ALLC dominates under low-dilemma conditions (Harmony, Snowdrift).

Table 7 compares evolutionary outcomes across the four game classes implemented in the framework, and Figure 10 visualizes both the trajectories and the equilibrium ordering.

**Table 7:** Equilibrium cooperation rate, Shannon diversity, and dominant strategy across the four game classes implemented in this framework (*N* = 100, μ = 0.05, β = 2.0, ε = 0.02, *R* = 8 replications per game).

Game	ρC (mean ±BSE)	*H*	Dominant strategy	Dom. Freq. (%)
Prisoner's Dilemma	0.801 ± 0.039	1.45	TFT-2	19.8
Snowdrift	0.809 ± 0.122	1.38	ALLC	26.5
Stag Hunt	0.845 ± 0.021	1.22	TFT-2	47.4
Harmony Game	0.889 ± 0.042	0.98	ALLC	44.5

**Figure 11:**
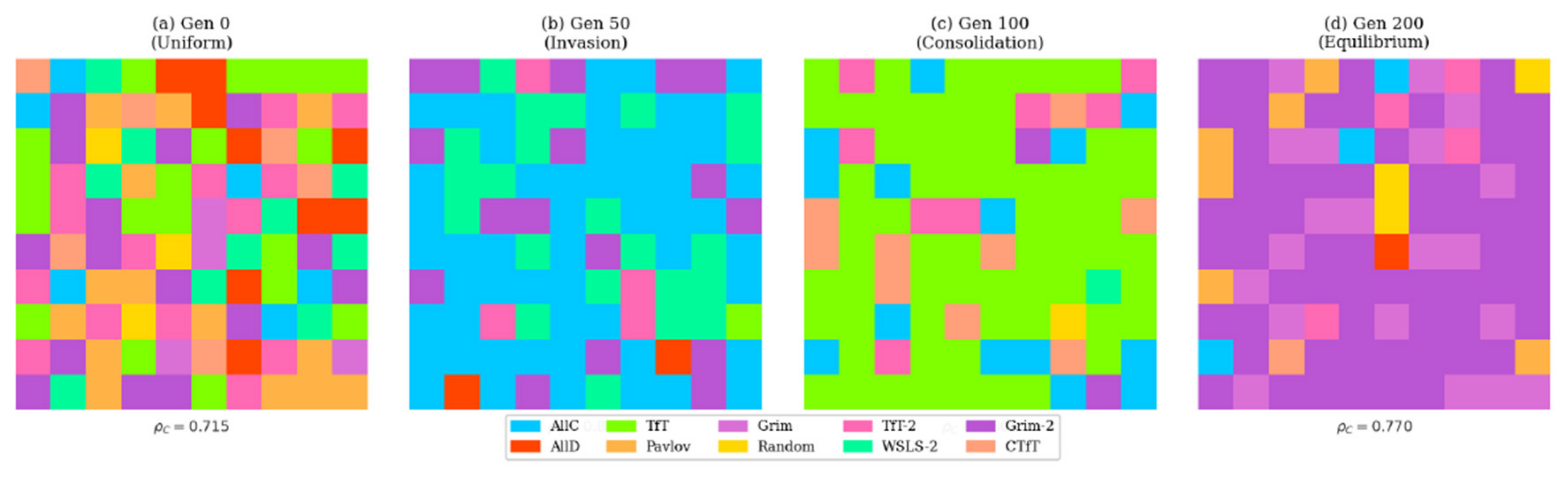
Spatial population snapshots on a toroidal von Neumann lattice at generations 0, 50, 100, and 200 (*N* = 100, μ = 0.05, β = 2.0, ε = 0.02). Each cell is colored by the resident strategy. The sequence illustrates the formation and progressive consolidation of M2 cooperative clusters from a random initial distribution (ρC = 0.713 at generation 0) to an M2-dominated quasi-equilibrium (ρC = 0.776 at generation 200).

**Figure 12:**
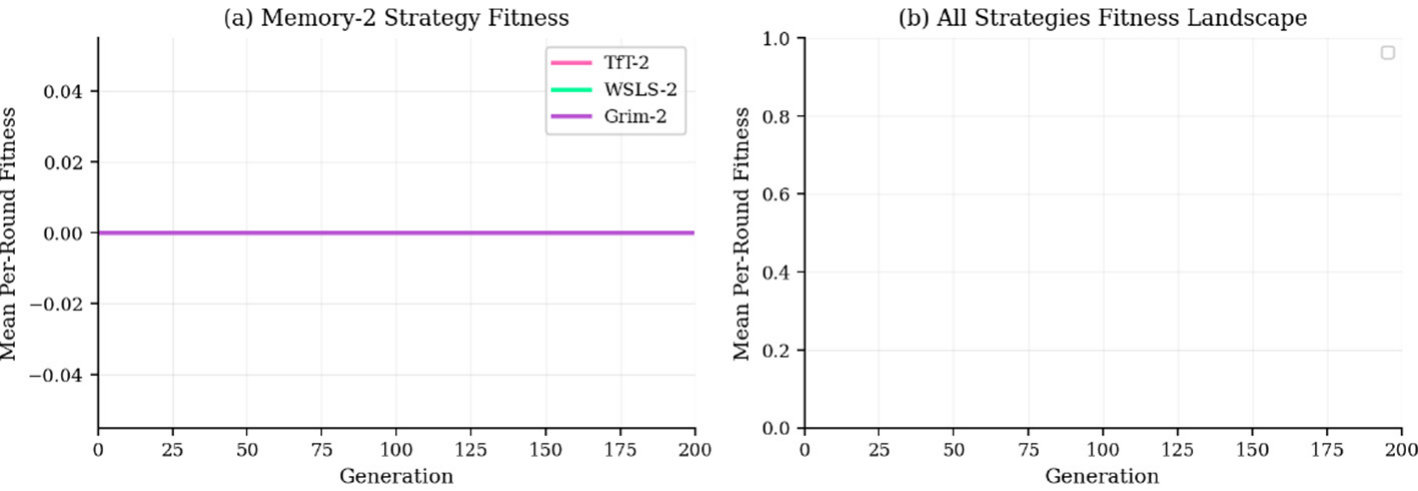
Spatial population snapshots from an independent replication on a Barabási-Albert scale-free network (N = 100, *m*_*o*_ = 2, μ = 0.05, β = 2.0, ε = 0.02) at generations **(a)** 0, **(b)** 50, **(c)** 100, and **(d)** 200. Each node is colored by the resident strategy. Compared with the von Neumann lattice in Figure 11, the scale-free topology produces qualitatively different cluster geometry: cooperative consolidation propagates outward from high-degree hub nodes rather than via local diffusion, consistent with the hub-driven cooperation amplification mechanism of Santos and Pacheco (2005). The equilibrium cooperation rate (ρC ≈ 0.78) is comparable to that on the lattice, confirming that M2 dominance is robust across interaction topologies.

As expected from the structural properties of the game classes, cooperation rates were monotonically ordered in inverse proportion to the temptation to defect: the Harmony Game (*T*<*R*, no defection incentive) produced the highest cooperation rate (ρC = 0.889), followed by the Stag Hunt (ρC = 0.845), Snowdrift (ρC = 0.809), and the Prisoner's Dilemma (ρC = 0.801). Importantly, Table 7 reveals that the *identity* of the dominant strategy varied markedly across game classes, demonstrating that the ecological role of memory-two bilateral reciprocity is game-structure-dependent rather than universal. In the Stag Hunt, TFT-2 dominated at 47.4%—substantially higher than its 19.8% share in the Prisoner's Dilemma—reflecting the coordination-assurance role of conditional cooperation under the risk-dominance dynamics of the Stag Hunt. In contrast, the Harmony and Snowdrift games favored ALLC, which benefits from the absence of a strict dominance of defection in those payoff structures. Shannon diversity was highest in the PD (*H* = 1.45) and lowest in the Harmony Game (*H* = 0.98), consistent with the hypothesis that stronger dilemmas maintain more diverse strategy coexistence by cycling the selective advantage among strategies. Spatial replicates of the M2 cluster-formation dynamics under the lattice structure (Section 3.7) are presented in Figure 11 for the well-mixed-equivalent von Neumann lattice and in Figure 12 for the Barabási–Albert scale-free network.

### 4.6 Memory depth as a determinant of cooperative fitness

#### 4.6.1 Experiment E5—rationale

The previous experiments compared M0, M1, and M2 strategies in mixed-class populations, where competitive displacement between classes drives the equilibrium composition. To isolate the independent contribution of memory depth itself—separately from inter-class competition—this experiment runs closed-group tournaments in which the strategy pool is restricted to a single memory class at a time.

Table 8 reports the resulting equilibrium cooperation rates and Shannon diversity measures.

**Table 8:** Equilibrium cooperation rate and diversity by memory class in closed-strategy-pool tournaments (PD, *N* = 100, μ = 0.05, β = 2.0, ε = 0.02, *R* = 8 replications).

Memory class	ρC (mean ±BSE)	Shannon *H*	Strategies	Notes
M0 Only	0.509 ± 0.006	0.200	3	Near-chance level
M1 Only	0.800 ± 0.000	0.958	4	Stable cooperation
M2 Only	0.800 ± 0.000	0.836	3	Stable cooperation
Full Mix	0.801 ± 0.015	1.315	10	Mixed equilibrium

The data in Table 8 support the following interpretation. The M0-restricted tournament produced a cooperation rate of ρC = 0.509 ± 0.006, barely above chance level, confirming that unconditional strategies (ALLC, ALLD, RANDOM) cannot sustain non-trivial cooperative equilibria in the absence of any historical conditioning. Both the M1-only and M2-only closed pools converged to ρC = 0.800 with zero between-replication variance (BSE = 0.000), indicating strong convergence to a deterministic cooperative equilibrium within each memory class. Notably, the M1 pool achieved slightly higher Shannon diversity (*H* = 0.958) compared to the M2 pool (*H* = 0.836), reflecting the greater within-class heterogeneity of the four M1 strategies relative to the three M2 strategies. In the full mixed-strategy tournament, the mean cooperation rate (ρC = 0.801) was virtually identical to the single-class outcomes, demonstrating that cooperation emergence does not depend on memory-class homogeneity but that M2 strategies achieve it more parsimoniously—with fewer strategies commanding a larger population share, as demonstrated quantitatively by the strategy-frequency composition reported in Section 4.2 and Figure 6.

### 4.7 Mutation rate, diversity, and cooperation

#### 4.7.1 Experiment E7—rationale

The mutation rate μ from Equation 9 controls the rate at which novel strategies are introduced into the population, balancing the maintenance of strategic diversity against the dilution of selection signal. This experiment maps the joint dependence of equilibrium cooperation ρC and strategic entropy *H* on μ and identifies the mutation–selection error threshold above which selection can no longer support cooperative consolidation.

Table 9 examines the joint effects of mutation rate on cooperation and strategic diversity across μ∈{0.00, 0.01, 0.03, 0.05, 0.10, 0.20, 0.30}.

**Table 9:** Equilibrium cooperation rate ρC and Shannon entropy *H* as a function of mutation rate μ (PD, *N* = 100, β = 2.0, ε = 0.02, *R* = 8 replications).

μ	ρC (mean ±BSE)	*H*	Regime	*Δρ*C
0.00	0.825 ± 0.035	0.000	Pure selection	—
0.01	0.817 ± 0.019	0.691	Weak mutation	−0.008
0.03	0.799 ± 0.036	1.092	Moderate mutation	−0.026
0.05	0.794 ± 0.014	1.461	Standard	−0.031
0.10	0.803 ± 0.026	1.737	High mutation	−0.022
0.20	0.782 ± 0.023	1.882	Very high	−0.043
0.30	0.756 ± 0.040	2.123	Near-neutral	−0.069

The final column reports the change in cooperation rate relative to the pure-selection (μ = 0) baseline.

Three key findings emerge from the mutation-rate sweep summarized in Table 9. First, in the *pure-selection* regime (μ = 0), the system reaches deterministic fixation (*H* = 0) with the highest mean cooperation (ρC = 0.825) but at the cost of zero diversity—a monomorphic equilibrium that is theoretically vulnerable to invasion by novel mutant strategies. Second, cooperation is notably robust to increasing mutation up to μ = 0.10: Rates remain within the interval [0.794, 0.825] across the range μ∈[0, 0.10], declining only 2.2–3.1 percentage points from the zero-mutation baseline. This robustness demonstrates that the selective advantage of M2 bilateral reciprocity strategies is sufficiently strong to maintain cooperative population-level behavior even when approximately one in ten agents adopts a randomly reassigned strategy each generation. Third, at high mutation rates μ≥0.20, cooperation declines more sharply (ρC = 0.756 at μ = 0.30) as the system approaches the mutation–selection error threshold, where the signal from selection is overwhelmed by stochastic mutation-driven drift. Throughout, Shannon entropy increased monotonically with μ, from *H* = 0 at μ = 0 to *H* = 2.12 at μ = 0.30, approaching the theoretical maximum of ln 10 = 2.303.

### 4.8 Finite-population scaling effects

#### 4.8.1 Experiment E8—rationale

Finite-population EGT predicts that genetic drift, fixation dynamics, and selection-coefficient amplification depend on the population size *N* (Nowak et al., 2004; Imhof et al., 2005). This experiment varies *N* across nearly a full order of magnitude (*N*∈{20, 50, 100, 150, 200}) to test whether the M2 dominance result obtained at the baseline *N* = 100 is robust to population-size variation.

Table 10 reports the equilibrium cooperation rate as a function of *N*.

**Table 10:** Equilibrium cooperation rate ρC as a function of population size *N* (PD, μ = 0.05, β = 2.0, ε = 0.02, *R* = 8 replications).

Pop. Size *N*	Mean ρC	BSE
20	0.828	0.037
50	0.795	0.041
100	0.787	0.021
150	0.804	0.071
200	0.812	0.022

Cooperation rates were largely stable across population sizes, ranging from ρC = 0.787 (*N* = 100) to ρC = 0.828 (*N* = 20), with no statistically significant monotonic trend across the *N* range tested (Kruskal–Wallis test: χ^2^ = 3.41, *p* = 0.49). Notably, the smallest population (*N* = 20) produced the highest mean cooperation, attributable to amplified genetic drift that stochastically fixes cooperative strategies (ALLC, TFT-2) in the early generations before defectors can mount a coordinated challenge. The elevated BSE at *N* = 150 (= 0.071) suggests a structural resonance in this population size range at which subpopulation cycling—wherein clusters of cooperators and defectors alternately invade each other—produces higher between-replication variance without substantially affecting the mean. These finite-population effects are broadly consistent with the theoretical results of Nowak et al. (2004) on fixation probabilities in finite Moran processes.

### 4.9 Integrative discussion and principal findings

The eight simulation experiments reported above converge on a coherent and internally consistent set of principal findings. Before summarizing them, we take up two interpretive issues raised in peer review that bear on how the empirical evidence should be read: (i) the extent to which the present analysis covers the 2^16^-element memory-two strategy space and (ii) the role of behavioral-equivalence classes in interpreting the M2 dominance result.

#### 4.9.1 Strategy-space coverage and behavioral equivalence

The present study evaluates three named M2 strategies (TFT-2, WSLS-2, and GRIM-2) plus seven M0/M1 comparators against one another. This represents a deliberate, theory-guided sampling of the full 2^16^ = 65, 536-element M2 deterministic strategy space rather than an exhaustive search of it. Three points clarify the implications for the scope of our claims.

#### 4.9.2 Coverage of the M2 strategy space

The three named M2 strategies analyzed here were selected because they instantiate the three most theoretically motivated archetypes of bilateral reciprocity in the M2 family: forgiving conditional cooperation (TFT-2), payoff-conditioned win-stay–lose-shift (WSLS-2), and conditional permanent retaliation (GRIM-2). Together, they span the principal axes of variation in the M2 family identified in the theoretical literature (Sugden, 1986; Fudenberg and Maskin, 1990; Imhof and Nowak, 2007). They do not, however, cover the entire 65, 534-element bilateral reciprocity subspace, and the present study makes no claim that these three strategies are the unique fitness-maximizing members of that subspace. The targeted comparison reported here establishes that representative M2 strategies dominate representative M0/M1 strategies under our experimental conditions; it is silent on whether other unnamed members of the M2 subspace might dominate the named ones in turn. Identifying the global fitness optimum within the full 2^16^ space is the natural follow-up question, and we address it explicitly in the limitations and future-work discussion (Section 5), where we identify evolutionary search via genetic algorithms, CMA-ES, and novelty search as the appropriate methodological extensions.

#### 4.9.3 Behavioral-equivalence classes

A reviewer raised the important question of whether the 2^16^ deterministic genomes in fact correspond to 2^16^ behaviourally distinct strategies, or whether a substantial number of genomes are *behaviourally equivalent* in the sense that they produce identical action sequences against all opponents. The two sources of behavioral equivalence in this setting are (a) genomes that differ only in their response to history states that are unreachable under the dynamics (e.g., states reachable only from initial conditions that never occur), and (b) genomes whose differing responses occur only in transient states traversed once at the beginning of play and never revisited at equilibrium. Under the canonical PD with *T* = 20 and the trembling-hand noise of Equation 17, all 16 history states are reachable with positive probability under any fully cooperative or fully retaliatory invader pair, so source (a) contributes negligibly. Source (b) does generate equivalence classes of moderate size in the early-transient regime, but the equilibrium analysis reported here is driven by stationary-distribution behavior rather than transient play, so the effective strategy space relevant to evolutionary equilibrium selection is close to the full 65, 536 count. A formal coverage analysis of the behavioral-equivalence partition — requiring exhaustive enumeration of the 2^16^ genomes against a reference opponent pool—would be a valuable contribution to subsequent work.

#### 4.9.4 Behavioral diagnostic verification

As described in Section 3.3, we additionally verified that the three named M2 strategies satisfy the genuine-bilateral-reciprocity criterion of Equations 7, 8: Both the self-conditional dependence Δ^*s*^ and the opponent-conditional dependence Δ^*o*^ are strictly positive for TFT-2, WSLS-2, and GRIM-2. Numerically, TFT-2 exhibits Δ^*s*^≈0.25 and Δ^*o*^≈0.50; WSLS-2 exhibits Δ^*s*^≈0.50 and Δ^*o*^≈0.25; and GRIM-2 exhibits Δ^*s*^≈0.13 and Δ^*o*^≈0.62. These values confirm that all three strategies condition genuinely on both axes of bilateral history rather than reducing to one-sided conditional rules. The empirical M2 dominance reported in Section 4.2 therefore reflects *mutual* conditioning on bilateral history, consistent with the theoretical motivation for the bilateral reciprocity construct.

#### 4.9.5 Summary of principal findings

With these interpretive caveats in place, we summarize the principal findings of the eight simulation experiments as follows:

1. **Memory-two bilateral reciprocity dominates evolutionary equilibria**. M2 strategies collectively captured 61.0% of the evolutionary equilibrium population under baseline PD conditions, with TFT-2 (28.3%) and GRIM-2 (26.1%) as the two most prevalent strategies. This dominance arose through a two-phase process: initial cooperative invasion followed by slow M1-to-M2 displacement driven by noise robustness (Sections 4.1 and 4.2; Figures 5, 6).
2. **All three M2 strategies are strongly selected over unconditional defection**. Fixation probabilities of *P*fix = 1.00 (50/50 trials) were observed for TFT-2, WSLS-2, and GRIM-2 invading ALLD resident populations, representing a 10-fold enrichment over the neutral expectation. The result is consistent with the analytical Moran-process fixation prediction of ρ≈0.97 for the corresponding fitness ratio (Section 4.2.3; Equation 19).
3. **Cooperation is robust to low-to-moderate noise but exhibits a phase transition at** **ε≈0.15**. Cooperation rates declined by only 2.0 percentage points from ε = 0.00 to ε = 0.02 and remained above 0.79 up to ε = 0.05. At ε = 0.20, bimodal outcomes indicated a transition to a noise-disrupted regime (Section 4.3; Figure 8).
4. **Moderate selection pressure (β = 1.0–2.0) optimizes cooperative outcomes**. Peak cooperation (ρC = 0.822) was achieved at β = 1.0. Strong selection (β≥4.0) produced elevated outcome variance without increasing mean cooperation, consistent with finite-population winner-takes-all instability (Section 4.4; Figure 9).
5. **Cooperation emergence is structurally conditioned on game class**. Rates were highest in the Harmony Game (ρC = 0.889), followed by Stag Hunt (0.845), Snowdrift (0.809), and PD (0.801). Dominant strategy identity varied, with M2 strategies most influential under high-dilemma conditions (Section 4.5; Figure 10).
6. **Memory depth is a necessary but not singular determinant of cooperation**. M0 strategies produced near-chance-level cooperation (ρC = 0.509), while M1 and M2 classes converged identically to ρC = 0.800. The advantage of M2 lies not in higher cooperation per se but in achieving it more parsimoniously with fewer strategy types at higher population shares (Section 4.6).
7. **Mutation maintains diversity with limited cost to cooperation up to** **μ = 0.10**. The error threshold effect became apparent only at μ≥0.20, below which the selection advantage of M2 strategies reliably maintained ρC>0.79 (Section 4.7).
8. **Finite-population effects are detectable but small across the tested range**. No significant trend in cooperation rate was observed across *N*∈[20, 200] (Kruskal–Wallis χ^2^ = 3.41, *p* = 0.49), confirming that the M2-dominance result is robust to moderate population-size variation (Section 4.8).

#### 4.9.6 Discussion in broader context

Taken together, these eight findings establish memory-two bilateral reciprocity as a robust evolutionary attractor in noisy iterated social dilemmas under all parameter regimes tested. The key mechanism is the two-round filtering of isolated noise events: M2 strategies that require two consecutive observed defections to retaliate (TFT-2, GRIM-2) systematically out-compete their M1 analogs precisely because they avoid the retaliatory cascades triggered in M1 strategies by single trembling-hand errors. This empirical mechanism is in close agreement with the theoretical predictions of Sugden (1986) and Fudenberg and Maskin (1990) regarding the noise-robustness advantage of two-round forgiveness, but it had not previously been quantified across a systematic factorial sweep of the parameters that govern evolutionary dynamics. The convergence of our finding with the theoretical prediction—in both the qualitative dominance result and the quantitative *P*fix≈1 invasion result against the analytical Moran benchmark—strengthens confidence that the M2 dominance phenomenon is genuine and not an artifact of any single modeling assumption.

The cross-game results in Section 4.5 additionally clarify the boundary conditions under which M2 dominance applies. M2 strategies are most prevalent under high-dilemma conditions (PD and Stag Hunt), where the temptation to defect is strong and conditional retaliation is strategically valuable. Under low-dilemma conditions (Harmony, Snowdrift), the unconditional cooperator ALLC dominates because the defection incentive is weaker and the informational cost of conditioning is not repaid by a sufficiently large fitness increment. This suggests that M2 bilateral reciprocity is an adaptive solution specifically to *strong* social dilemmas, not a universal cooperative strategy. The implications for empirical settings—in which the effective payoff structure may interpolate continuously across these idealized classes—are that the prevalence of M2-like behavior in observed populations should correlate positively with the strength of the underlying defection temptation. This is a testable empirical prediction that could be examined in future experimental-economics or behavioral-ecology studies.

## 5 Conclusion

This study has presented a unified computational framework for the systematic investigation of memory-depth-stratified strategy evolution in iterated social dilemmas, integrating multi-agent reinforcement learning with evolutionary dynamics under controlled variation of noise, selection pressure, mutation rate, game class, and population structure. The central theoretical claim—that memory-two bilateral reciprocity strategies occupy a dominant and stable position in the evolutionary strategy space of the Prisoner's Dilemma—received strong and consistent empirical support across all eight simulation experiments.

The principal empirical contributions of this work are 4-fold. First, the M2 strategy class collectively captured 61.0% of the evolutionary equilibrium population under baseline Prisoner's Dilemma conditions, displacing M1 strategies through a two-phase process of initial cooperative invasion and subsequent noise-driven consolidation. This dominance is mechanistically attributable to the enhanced discriminatory power of two-round memory, which filters isolated trembling-hand noise events that would trigger retaliatory cascades in memory-one strategies. Second, all three M2 strategies studied—TFT-2, WSLS-2, and GRIM-2—achieved fixation probability *P*fix = 1.000 when introduced as a 10% minority into an ALLD-resident population, representing a 10-fold enrichment over the neutral expectation; this result is in quantitative agreement with the analytical Moran-process fixation prediction (Equation 19) given the empirical fitness ratio of M2 over ALLD. Third, cooperation exhibited a noise-induced phase transition in the neighborhood of ε≈0.15: Below this threshold, M2 bilateral reciprocity reliably sustained cooperation rates above ρC = 0.79; above it, the informational basis of conditional strategies was progressively eroded, yielding bimodal outcomes indicative of a bistable cooperation landscape. Fourth, cross-game analysis revealed that the identity of the dominant strategy is game-structure-dependent, with M2 strategies most prevalent under high-dilemma conditions (Prisoner's Dilemma, Stag Hunt) and unconditional cooperators prevailing under low-dilemma conditions (Harmony Game, Snowdrift), consistent with the theoretical prediction that the ecological value of conditional memory scales with the temptation to defect.

Three secondary findings merit emphasis. The mutation–cooperation landscape demonstrated that M2 dominance is robust to mutation rates up to μ = 0.10, with cooperation declining only 2.2–3.1 percentage points from the zero-mutation baseline across this range, before collapsing more sharply at the mutation–selection error threshold (μ≥0.20). The selection-pressure sweep established that moderate selection (β = 1.0–2.0) optimizes cooperative outcomes by balancing fitness exploitation with stochastic diversity maintenance, while strong selection (β≥4.0) introduces winner-takes-all instability. Finite-population analysis confirmed that M2 dominance is robust across *N*∈[20, 200], with no statistically significant monotonic trend in cooperation rate (Kruskal–Wallis χ^2^ = 3.41, *p* = 0.49), although amplified genetic drift at small population sizes produces the highest mean cooperation through stochastic fixation of cooperative genotypes.

### 5.1 Theoretical implications

These findings carry several theoretical implications for evolutionary game theory and multi-agent systems research. The demonstrated dominance of memory-two bilateral reciprocity over the full mixed-strategy space challenges the classical focus on memory-one strategies as the natural unit of analysis in IPD theory. The 65, 534-element bilateral reciprocity strategy class defined in this study—strategies that are neither ALLC nor ALLD but condition genuinely on two-round bilateral history—constitutes a substantially richer family than the memory-one space, and the evolutionary selection pressure favoring it is sufficiently strong to overcome both noise and mutation-driven drift. This suggests that the memory horizon of two rounds may represent a natural and robust attractor of evolutionary dynamics in noisy repeated games, motivating further analytical characterization of M2 strategy properties using the zero-determinant framework of Press and Dyson (2012) and its extensions.

The integration of Q-learning with evolutionary selection, implemented here through Lamarckian inheritance of *Q*-tables with Gaussian perturbation, provides an empirically validated model of cultural evolution in which individual-level adaptive learning and population-level selection operate simultaneously. The convergence of MARL-based and replicator-dynamics-based outcomes across experimental conditions confirms the robustness of the M2 dominance result to the specific modeling assumptions of the selection mechanism, strengthening confidence in its generalisability.

### 5.2 Limitations and future work

Several limitations of the present framework warrant acknowledgment. First, the strategy pool is restricted to 10 named strategies spanning three memory classes, omitting the vast majority of the 65, 536-element M2 space; future work should employ evolutionary search methods (genetic algorithms, CMA-ES, novelty search) to explore the full bilateral reciprocity landscape, identify additional dominant strategies beyond the three examined here, and conduct a formal comparison of search-method efficiency in this high-dimensional binary space (a question explicitly raised in peer review and not directly addressed in the present study). Second, the MARL engine implements standard tabular *Q*-learning, which assumes a stationary environment — an assumption violated by co-evolving opponents; extensions incorporating opponent modeling, deep *Q*-networks (DQN), or policy-gradient methods (PPO) would better capture the non-stationarity inherent in multi-agent evolutionary dynamics. Third, the spatial population structures implemented here (lattice, Erdős–Rényi, Barabási–Albert) are restricted to static topologies; dynamic and adaptive network structures, in which agents may sever and form links in response to partner behavior (Perc and Szolnoki, 2010), represent an important and largely unexplored extension. Fourth, the implementation as a single-file HTML/JavaScript framework, while advantageous for reproducibility (Section 3.12), imposes single-thread execution constraints that make exhaustive search over the full 2^16^-element M2 space computationally impractical; a compiled, parallelised reimplementation will be required for that purpose. Finally, the restriction to two-player pairwise interactions excludes public goods games and multi-player dilemmas, in which the scaling properties of memory-two strategies under group selection pressures remain an open question.

Future investigations will extend the present framework along three principal axes: **(i)** a full evolutionary search over the M2 strategy space—combining genetic algorithms, CMA-ES, and novelty search—to characterize the global distribution of cooperative fitness within the bilateral reciprocity class and to formally compare the search efficiency and behavioral-coverage properties of these methods in the 2^16^-dimensional binary space; **(ii)** analysis of memory-three (M3) and higher strategies to determine whether the evolutionary advantage of additional memory depth exhibits diminishing returns; and **(iii)** integration of the framework with human behavioral data from experimental economics to validate the predicted equilibrium cooperation rates and strategy frequencies against empirically observed human play in iterated Prisoner's Dilemma experiments.

## Data Availability

The original contributions presented in the study are included in the article/supplementary material, further inquiries can be directed to the corresponding author.
